# Fatty acid abnormalities in cystic fibrosis–the missing link for a cure?

**DOI:** 10.1016/j.isci.2024.111153

**Published:** 2024-10-11

**Authors:** Sławomira Drzymała-Czyż, Jarosław Walkowiak, Carla Colombo, Gianfranco Alicandro, Olav Trond Storrösten, Magnhild Kolsgaard, Egil Bakkeheim, Birgitta Strandvik

**Affiliations:** 1 Department of Bromatology, Poznan University of Medical Sciences, Poznan, Poland; 2 Department of Pediatric Gastroenterology and Metabolic Diseases, Institute of Pediatrics, Poznan University of Medical Sciences, Poznan, Poland; 3 Cystic Fibrosis Centre, Fondazione IRCCS Ca’ Granda Ospedale Maggiore Policlinico, and Department of Pathophysiology and Transplantation, University of Milan, Milan, Italy; 4 National Resource Centre for Cystic Fibrosis, Oslo University Hospital, Oslo, Norway; 5 CLINTEC, Karolinska Institutet NOVUM, Stockholm, Sweden

**Keywords:** Health sciences, Medicine, Medical specialty, Clinical genetics, Internal medicine, Respiratory medicine, Human genetics, Molecular biology

## Abstract

The care for cystic fibrosis (CF) has dramatically changed with the development of modulators, correctors, and potentiators of the CFTR molecule, which lead to improved clinical status of most people with CF (pwCF). The modulators influence phospholipids and ceramides, but not linoleic acid (LA) deficiency, associated with more severe phenotypes of CF. The LA deficiency is associated with upregulation of its transfer to arachidonic acid (AA). The AA release from membranes is increased and associated with increase of pro-inflammatory prostanoids and the characteristic inflammation is present before birth and bacterial infections. Docosahexaenoic acid is often decreased, especially in associated liver disease Some endogenously synthesized fatty acids are increased. Cholesterol and ceramide metabolisms are disturbed. The lipid abnormalities are present at birth, and before feeding in transgenic pigs and ferrets. This review focus on the lipid abnormalities and their associations to clinical symptoms in CF, based on clinical studies and experimental research.

## Graphical abstract

**Figure:**
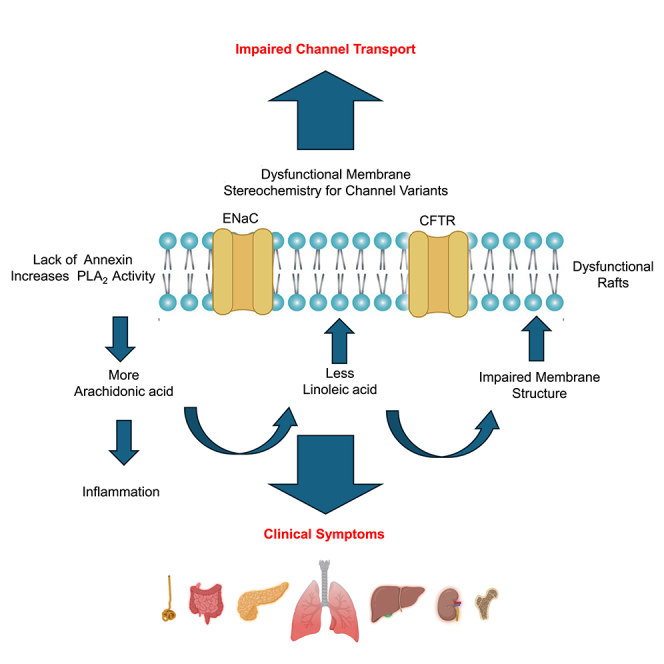

## Introduction

Although 35 years have passed since the gene for cystic fibrosis (CF), the cystic fibrosis transmembrane conductance regulator (CFTR), was identified and more than 2200 mutations have been registered (The Clinical and Functional Translation of CFTR (CFTR2); listed at http://cftr2.org), a cure for the disease has not yet been found. Only about 30% of gene variants of the CFTR, a chloride and bicarbonate channel, have been found clinically relevant for the CF disease. Despite extensive research in the latest decades, difficulties remain to fully explain the link between the channel and many of the clinical symptoms.^1^^,^^2^ It is also of interest that CF-like phenotypes have been produced in animal experiments by inhibition of nuclear liver X receptor (LXR)β,^3^^,^^4^ and by genetic variants of the epithelial sodium channel (ENaC),^5^ but the associations to CFTR are not fully explored.

New CFTR modulating therapies with protein chaperones (potentiators-correctors) have made great improvement in life for people with CF (pwCF), but are not available for all patients.^6^^,^^7^^,^^8^^,^^9^ The latest triple combination, elexacaftor-tezacaftor-ivacaftor (ETI; Kaftrio/Trikafta), has shown important clinical effects related to the most common mutation, Phe508del, with increase of lung function about 14% – much more than previous combinations – and with positive impact on the general clinical status.^10^^,^^11^^,^^12^^,^^13^^,^^14^^,^^15^^,^^16^^,^^17^ This is a large step forward, but can only help pwCF with certain mutations^18^^,^^19^ and has limited influence on the chronic inflammation.^20^^,^^21^^,^^22^^,^^23^^,^^24^ So far, some serious adverse effects have been reported,^16^^,^^25^^,^^26^^,^^27^^,^^28^^,^^29^^,^^30^^,^^31^^,^^32^ like liver failure^29^ and psychological disturbances^33^^,^^34^^,^^35^^,^^36^^,^^37^^,^^38^

New interest to the lipid disturbances in CF has emerged since the modulators of the CFTR, from Ivacaftor to the latest triple combination ETI, have been shown to interfere with the lipid metabolism, especially with phosphatidylcholine (PC) and ceramides, both in humans^39^^,^^40^^,^^41^ and in CF cell cultures.^41^^,^^42^^,^^43^^,^^44^ Table 1 summarizes studies about lipid interferences with the modulators so far. The cellular membranes are mainly built of phospholipids, cholesterol and ceramides.^45^ The physiology of proteins in membranes are highly dependent on the lipid structure, which varies between different tissues and different organelles.^46^^,^^47^^,^^48^^,^^49^^,^^50^ It is of interest that it has repeatedly been reported that PC, the dominating phospholipid in the outer cell membrane, has an increased turnover in CF.^51^^,^^52^^,^^53^^,^^54^^,^^55^ Using matrix-assisted laser desorption ionization time of flight (MALDI-TOF) technique with mass spectrometry, Guerrera et al.^56^ found that six PC and four lysophosphatidylcholine (LPC) molecules were significantly decreased in pwCF with differences between severe and mild phenotypes, corroborating clinical findings that plasma phospholipid profiles in mild phenotypes may not differ from those in healthy controls.^57^^,^^58^^,^^59^

**Table 1:** Recent update of lipid abnormalities in Cystic Fibrosis in relation to CFTR mutations and CFTR modulators

Reference	Tissue	Method/Modulator	Lipids	Metabolic effect
***In vitro* studies**				

Liessi et al.^60^	Human dF508del bronchial cells Review	Ivacaftor, Lumacaftor, Orkambi, ETI	Ceramides, sphingomyelins, phospholipids, diacylglycerols	ETI, more than other modulators, blocks formation of ceramides from dihydroceramides and downregulates major lysoPC independent of CFTR variants. Associated with less apoptosis.
Abu-Arish et al.^44^	Human bronchial dF508del and S13F cells	Detergents, Lumacaftor Spectroscopy Immunostaining Short-circuit current	Membrane lipid order in rafts	CFTR clustering and membrane integration are dependent of membrane lipid order. Defective clustering restored by modulators and detergents.
Abu-Arish et al.,^61^	Human bronchial cells	Spectroscopy Confocal microscopy	Cholesterol	Different clustering of CFTR in membranes depending on cholesterol concentration
Abu-Arish et al.,^62^	Human dF508del bronchial cells	Live cells imaging, Spectroscopy Immunology Ussing chambers VIP, Carbacol	Cholesterol, ceramides	CFTR surface expression influenced by ceramide-rich platforms aggregated from clusters at stimulation by VIP and carbachol triggered by ASMas.
Liessi et al.^63^	Human primary bronchial epithelial cells from dF508del, and from M/Min mutations	ETI MS, High pressure chromatography	Ceramides	ETI impacts conversion of dihydroceramides to ceramides independent of CFTR variants. Suggest neurological risk factor?
Schenkel et al.^64^	Unilamellar vesicles of phosphatidylcholine (POPC) with CFTR TM loops 3/4 and intervening extramembrane loop ± cholesterol	Lumacaftor Fluorescence microscopy	Cholesterol, Protein action	Lumacaftor shields protein from lipid environment improving function/configuration, independent of cholesterol

**Combined studies**				

Veltman et al.^42^	Air-liquid interface in pulmonary cells from CF mice lungs, CF-KO pigs and human CF airway epithelium cultures	Orkambi, ETI Fenretinide LC/MS, protein inflammatory markers, Ussing chambers Inflammatory markers	Fatty acids, ceramides, cytokines	Improvement of lipid pattern by modulators but persistent inflammation markers and oxidative stress markers. High *n*-6/*n*-3 ratio (AA/DHA), and low ceramide levels improved but not normalized by fenretinide and ETI, resp.
Centorame et al.,^65^	F508del^tm1EUR^ mice, (only 3 mice in each group) *in vitro* cell lines	ETI Fenretinide GC	Fatty acid, lipid profiles	Increase of EPA and DHA, decrease of saturated fatty acids. Fenretinide better effect than ETI on lung function, histology, inflammation; combination of drugs similar to fenretinide or even better. Glucosylation of defective CFTR increased by fenretinide and combination with ETI, but not by only ETI.
Bae et al.^66^	*CFTR*^*+/+*^ vs. *CFTR*^*−/−*^ piglets Comparisons 8 vs. 8 animals, within 12 h from birth Cell culture	Arteriovenous metabolomics in different organs LC/MS	Fatty acids, mono-di- and triglycerides,	Increased nonesterified long-chain fatty acids and decreased LA in CF piglets

**Clinical studies**				

Petersen et al.^31^	Blood samples from pwCF Retrospective, observational study, 134 CF adults (female 46%)	ETI	Cholesterol Lipoproteins	Increase of Cholesterol, LDL-C, obesity and blood pressure
Despotes et al.^32^	Blood samples from pwCF Retrospective observational study, 41 CF adults (female 66%), aged 16–73 years	ETI	Cholesterol lipoproteins	Increase of Cholesterol, LDL-C, TC/HDL-C, more in family history of CVD, less in CFLD
O'Connor and Seegmiller^39^	Blood samples from pwCF G5551D variant Prospective, observational study, 20 children pwCF (female 50%), 20 adults pwCF (female 80%))	Ivacaftor	AA, PGE-M, *n*-3 fatty acids	Decrease of AA and its metabolite PGE-M. No change in *n*-3 fatty acids.
Zardini Buzatto et al.^40^	Blood samples from pwCF 42 pwCF (female 60%), aged – 12–34 years.	HPLC and LC-MS Databases	Ceramides, sphingomyelins, fatty acids, diacylglycerols	Lipid pattern, especially phosphatidic acids and diacylglycerols, differed by different mutation classes. Five oxidized lipids correlated to FEV_1_
Lonabaugh et al.^67^	Human blood from different variants of pwCF Retrospective chart review study, 128 adults pwCF (female 52%)	ETI	Cholesterol, Lipo-proteins	ETI increases Cholesterol, LDL-C and HDL-C
Patel et al.^68^	Human blood from homo and heterozygotes of pwCF dF508del Retrospective study, 137 adults (*n* = 110) and adolescents (*n* = 26) (female 40%))	ETI	Cholesterol, lipoproteins	ETI increases Cholesterol, LDL-C, HDL-C, S levels of vitamins A and D
Tindall et al.^69^	Blood samples from pwCF 12 children (female 60%), age 4–20 months.	Ivacaftor	Fatty acids	Ivacaftor increased total saturated fatty acids, mead acid, palmitic acid and docosatetraenoic acid, indicating EFAD.
Tindall et al.^70^	Blood samples from pwCF homozygous for F508del Prospective longitudinal study, 21 children (female 61%), aged 2–5.9 years	Orkambi	Fatty acids, fat soluble vitamin	Lumacaftor/Ivacaftor increased S- retinol. No change in plasma total fatty acids but decrease of ALA and DHA.
McDonald et al.^71^	Serum from 142 pwCF (4 months-18 years) homo- and heterozygotes for dF508	Ivacaftor, Orkambi Symdeko	Fatty acids, lipoproteins	No influence of modulators on EFA deficiency, but some influence on LDL, HDL, VLDL and triglycerides
Slimmen LJM et al.^72^	Bronchoalveolar lavage fluid from 46 pwCF aged 1–5 years.	Lipid mediators at HPLC-tandem MS at 65 samples 8/65 on Orkambi	Lipid mediators of linoleic acid and arachidonic acid	No influence by modulators. Increase of AA derivatives and decrease of LA derivatives in relation to neutrophiles and pulmonary damage.
Yuzyuk et al.^73^	Blood samples from 153 pwCF (mean age 10.1 ± 4.7 years).	Ivacaftor, Orkambi, Symdeko, ETI. 65% on modulators for >1 mnth	Lipoproteins	Modulators increased HDL cholesterol, lowered LDL, VLDL and triglycerides comparing to controls.

AA, arachidonic acid; ALA, alpha linolenic acid; ASMas, acid sphingomyelinase; C, cholesterol; CFLD, cystic fibrosis liver disease; CFLD, cystic fibrosis liver disease; CFTR, Cystic fibrosis transmembrane conductive regulator; CVD, cardiovascular diseases; DHA, docosahexaenoic acid; EFAD, essential fatty acid deficiency; ETI, Ivacaftor+elexacaftor+tezacaftor; EPA, eicosapentaenoic acid; FEV_1_, forced expiratory volume in 1 s; HPLC, high-pressure liquid chromatography; LA, linoleic acid; LC, Liquid chromatography; L/HDL, low/high-density lipoprotein; MS, masspectrometry; TM, transmembrane; Orkambi, Ivacaftor+Lumacaftor; Symdeko, Ivacaftor+Tezacaftor.

Linoleic acid (LA, 18:2*n*-6) is an important component of membrane phospholipids.^74^^,^^75^^,^^76^^,^^77^ Thus the LA deficiency in CF, which has been repeatedly described since more than 60 years,^78^ and verified in hematological elements as well as in different other tissues,^79^^,^^80^^,^^81^ can be one factor associated with the disturbed channel localization and activities. The composition of fatty acids in membranes is important for the membrane function, influencing transports and metabolism in all cells and tissues,^45^^,^^46^^,^^76^^,^^82^^,^^83^^,^^84^^,^^85^ and the local tissue balance is not always reflected in the blood.^85^^,^^86^ The essential fatty acids (EFA), their long-chain derivatives and corresponding lipid mediators of the *n*-6 and the *n*-3 series, are also important for the inflammatory homeostasis,^87^ which is seriously disturbed in CF.^21^^,^^88^ Since channels mainly act in membrane rafts, markedly influenced by ceramides^63^^,^^89^^,^^90^^,^^91^^,^^92^^,^^93^^,^^94^^,^^95^^,^^96^ and cholesterol, the disturbances in these lipids are also important for the channel functions.^97^^,^^98^^,^^99^^,^^100^^,^^101^^,^^102^^,^^103^^,^^104^^,^^105^^,^^106^^,^^107^^,^^108^ The importance of membrane lipids for cell functions and the limitations of modern modulator therapy, hitherto not shown to improve the characteristic lipid abnormalities in CF, are the facts behind this review. The aim is to describe the associations between the lipid divergences, focusing on the *n*-6 fatty acids, and the clinical symptoms, as a challenge to optimize the treatment of pwCF.

## Lipid abnormalities

### Fatty acids

The most consistent EFA disturbance in CF fatty acid metabolism is the low concentration of LA in blood; in total lipids, cholesterol esters, phospholipids, platelets and red cell membranes.^58^^,^^78^^,^^79^^,^^80^^,^^81^^,^^109^^,^^110^^,^^111^^,^^112^^,^^113^^,^^114^^,^^115^^,^^116^^,^^117^^,^^118^^,^^119^^,^^120^^,^^121^^,^^122^^,^^123^^,^^124^ Other tissues are more seldom examined, but show marked deficiency, also in comparison with the plasma levels.^79^^,^^80^^,^^81^^,^^125^ Interestingly, CF-knock out (KO) pigs and ferrets have low LA at birth before feeding and the typical lipid changes are also found in the liver of newborn CF pigs.^66^^,^^126^ Arachidonic acid (AA, 20:4*n*-6) concentrations varies, but the ratio AA/docosahexaenoic acid (22:6*n*-3, DHA) is consistently increased.^57^^,^^81^^,^^119^^,^^127^^,^^128^^,^^129^ Analyses of the arteriovenous ratio in different organs of newborn CF piglets showed marked increase of AA.^66^ The long-chain polyunsaturated fatty acid (LCPUFA) of the omega-3 series, is often decreased in plasma and tissues, especially in patients with CF related liver disease (CFRLD).^130^^,^^131^ Endogenously synthesized fatty acids, like palmitoleic acid (16:1*n*-7, PA), oleic acid (18:1 *n*-9,OA) and eicosatrienoic acid (20:3*n*-9, Mead acid) are increased in CF,^57^^,^^110^^,^^132^^,^^133^ reflected in increase of the fatty acid transforming enzymes in CF cells.^54^ Parts of the typical fatty acid profile have been suggested as markers for the CF diagnosis, e.g., the classical EFA index (Mead acid/AA or expressed triene/tetraene (T/T) ratio),^133^ the ratio PA/LA^113^ and the product DHA x LA.^134^ Algorithms to relate symptoms to low LA concentrations and high AA release, respectively, were suggested for the gastrointestinal tract and the airways^135^^,^^136^ and have gained support in recent studies, as discussed below.

The LA concentration is usually inversely correlated to AA, both in CF,^137^ some other pathological conditions and in healthy individuals.^138^^,^^139^^,^^140^^,^^141^ In severe LA deficiency as in malnutrition, not related to CF, AA is generally decreased,^142^ which observation might have impact also in malnourished pwCF.^143^ The LA deficiency in CF varies in relation to CFTR variants and to dietary intake.^57^^,^^119^^,^^127^^,^^144^ Older studies have shown that obligatory heterozygotes for the CFTR variants had slightly different fatty acid profile compared to controls, suggesting a milder fatty acid abnormality.^110^^,^^115^ as also indicated in pwCF with pancreatic sufficiency.^114^^,^^117^ A recent comprehensive study examining the lipid pattern between different classes of mutations confirmed significant differences in relation to the symptomatic spectrum.^40^

The role of *n*-3 fatty acids has been extensively discussed after the publication by Freedman et al.^145^ showing an association between low DHA and abnormal intestinal and pancreatic morphology in CF mice, which improved by DHA supplementation. These improvements were not confirmed in a long-time running study of DHA supplementation in CF mice,^146^ but showed protection from liver damage, supporting clinical observations of associations between low DHA and liver disease in CF.^130^^,^^131^ Many *n*-3 supplementation studies in pwCF showed increase of plasma concentrations without clinical improvements,^147^^,^^148^ but a few studies showed a slight decrease of inflammation markers.^148^^,^^149^ In animal studies the DHA concentration varies,^150^ suggesting that the *n*-3 abnormality might be secondary, possibly related to long-term increased PC synthesis by the internal pathway.^108^^,^^151^ Although a basic disturbance of DHA in CF pathology is controversial, achievements of adequate concentrations would be of importance because specialized pro-solving mediators (SPM) of the long-chain *n*-3 fatty acids have a modulatory effect on inflammation.^152^^,^^153^

### Ceramides

The role of sphingolipids are multifactorial, and especially the impact of ceramides on the raft process in membranes might be of importance for the action of CFTR.^154^ An imbalance with an increased ratio between very long chain and long chain ceramides influencing the stability of rafts in cell membranes have been suggested to influence the sensitivity to Pseudomonas infection in pwCF^155^ and have been connected with increase of inflammatory cytokines.^156^ Type of sphingolipids are important for protein function and the organization of rafts.^157^ Their roles in the pathophysiology of CF has mainly been focused on the airways but conclusive results have been restricted due to non-specific antibody results. Interesting results are provided by the supplementation of fenretinide, a synthetic retinoid (N-(4-hydroxy-phenyl retinamide) improving the ceramide balance^158^ influencing many functions, e.g., in the lungs and improving the AA/DHA ratio.^97^ Further improvement in lung function and fatty acid status was found in CF KO mice and in Phe508del(tm1EUR) mice by a combined therapy of fenretinide and the modulator ETI.^65^^,^^159^

### Cholesterol

Cholesterol is low in serum of pwCF and in CF animal models but usually increased intracellularly.^98^^,^^99^^,^^103^^,^^160^ A Polish study found significant differences between patients with CFTR mutations associated with severe or mild disease, which differences also referred to other sterols.^161^ The cholesterol concentration is related to CFTR and fatty acids and its function to stabilize rafts makes its influence on the function of CFTR and other membranous proteins important,^67^^,^^101^^,^^104^ especially in the context of disturbed ceramide metabolism, also important for raft building. Its significance in the pathophysiology of CF from a clinical point of view has been limited, but it is interesting that the latest modulators have a great impact on the LDL-cholesterol (Table 1). The oxidation of cholesterol to keto-cholesterol is usually increased,^162^ which might be related to the generally increased oxidation in CF. The finding of a CF like disease in the LXRβ −/−mice might be related to the impact of defective LXR metabolism on the cholesterol and fatty acid balances.^4^^,^^163^^,^^164^^,^^165^^,^^166^

## Associations between clinical symptoms and fatty acid balance

**Figure 1:**
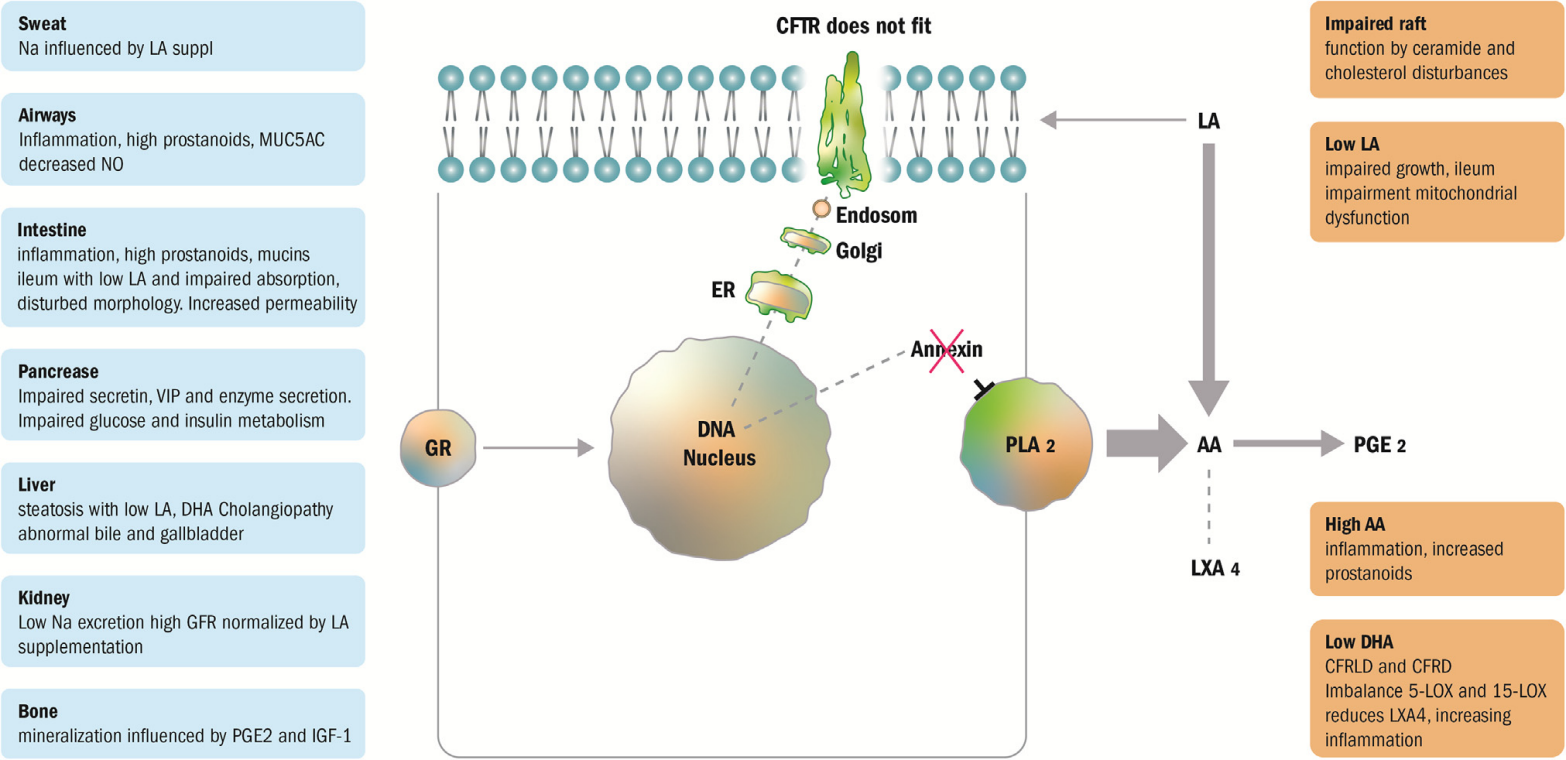
A basic simplified overview of the hypothetical relation between fatty acid abnormalities in relation to CFTR and clinical implications CFTR must fit in the membrane for normal function. CFTR variants are processed in the cell and abnormal CFTR are sorted in the endoplasmic reticulum, where most aberrant CFTR is withdrawn, and not all reach the membranes where the localization or function may be impaired in the context of membrane lipids. In normal cells glucocorticoids interfere with the annexin synthesis, which possibly by phosphorylation regulate PLA_2_ activity by inhibition both of its release of arachidonic acid and activity of COX 2 for PGE_2_ release. In CF down-regulation of annexin synthesis increases arachidonic acid release with subsequent increasing synthesis from linoleic acid. Thereby linoleic decreases in the membranes interfering with the membrane stereochemistry hampering CFTR activity, and thus impairing the action between CFTR and membranes. Fatty acid composition differs in membranes and organelles, whereby the defective CFTR transfer/function might occur in different compartment localizations in cells and organs. Defective metabolism of ceramides and cholesterol interferes with the raft building (not shown), where the CFTR have its action. Low DHA have been described in cystic fibrosis liver disease (CFRLD) and may be connected with cystic fibrosis related diabetes (CFRD) and may be related to low LXA_4_. The boxes to the left summarize lipid related processes described in the section “associations between clinical symptoms and fatty acid balance”. GR, Glucocorticoid Receptor; AA, arachidonic acid; ER, endoplasmic reticulum; LA, linoleic acid; PLA_2_, phospholipase A_2,_; PGE_2_, prostaglandin E_2_; LXA_4,_ lipoxin A4. Differences in arrow size reflect relative transfer activity.

An overview of the symptomatology, which can be related to the fatty acid disturbances in CF, is briefly summarized in Figure 1 and is described for the mainly involved tissues below.

### Sweat test

Sweat test is still the most important diagnostic test for CF.^167^^,^^168^ High chloride concentration is associated with genotypes with more severe clinical phenotype.^169^^,^^170^ Sweat chloride concentration are generally reduced by treatment with potentiators-correctors,^11^^,^^171^^,^^172^^,^^173^ but not by long term fatty acid supplementation.^174^ On the other hand, LA supplementation was associated with a reduction of sodium in sweat,^123^^,^^175^^,^^176^^,^^177^ which might be related to ENaC, active in sweat glands.^178^^,^^179^ (*cf* the kidney section below).

### Growth

Impaired growth is common in CF and certainly related to several factors. It is the reason for the general recommendations of very high energy intake.^180^^,^^181^^,^^182^ Recommendations of very high fat intake without specifying type of fat have resulted in increasing consumption of saturated fatty acids^183^ and problem with obesity^184^^,^^185^ increasing the risk for cardiovascular diseases.^32^^,^^186^ This is not seen in pwCF with supplementation of LA, who even can keep normal weight without increased energy intake.^187^^,^^188^ Thus, the classical and often first clinical symptom of EFA deficiency (EFAD) is impaired growth,^189^^,^^190^ which has been observed in pwCF already at birth.^191^^,^^192^ A series of studies from the neonatal screening program for CF in Wisconsin have strongly contributed to the knowledge about *the impact of LA on early growth.*^120^^,^^125^^,^^144^^,^^193^ This group has shown that at diagnosis 80% of pwCF with meconium ileus had LA deficiency, as indicated by high Mead acid and its T/T ratio at diagnosis, and that similar pathology were seen in around 50% of newborn pwCF without meconium ileus.^120^ A relative improvement occurred during infancy, but again the markers deteriorated in late toddler age – prepuberty, which coincides with the well documented dip in growth of pwCF, illustrated in both the European and North American CF patient registries.^194^^,^^195^ The series of papers from the Wisconsin CF Neonatal Screening program have shown that adequate body weight up to 2 years of age was dependent on both energy intake and adequate plasma concentrations of LA.^120^ Persistently low plasma concentration of LA despite high energy intake resulted in an OR of 7.46 for low growth compared to children with persistently adequate LA concentration and high energy intake, also after adjustment for the initial LA concentrations.^144^ By maintaining high LA intake above 5% of total energy intake, both the high energy and the LA concentration increased the possibility to catch up weight at 2 years of age (OR 30.6, *p* = 0.002). The authors concluded that “maintaining normal plasma LA for a prolonged period in addition to sustaining a high energy intake may be important in facilitating adequate weight gain”.^144^ The “early responders” also required lower energy intake to maintain adequate growth.^196^ In a follow-up study the Wisconsin group found that the early growth response was associated with better growth and lung function up to 12 years of age and also that pwCF with meconium ileus, who initially had a more severe LA deficiency, continued to be much worse off regarding growth.^193^^,^^197^ Some studies providing LA for shorter times, also reported an improvement in growth.^180^^,^^198^

An association between low LA concentration and poor growth can have different explanations. PwCF have *low IGF-1 concentrations,*^199^^,^^200^^,^^201^^,^^202^^,^^203^ also found in newborn CF pigs.^203^ Low levels of IGF-1 correlated to impaired growth,^202^^,^^204^ and to low lean body mass (LBM) in pwCF^205^ and in transgenic CF mice and rats.^206^^,^^207^ Rosenberg et al.^206^ reported low growth hormone (GH) levels in CF mice, which might be expected since IGF-1 is a major stimulator for GH. However, the relation between GH and IGF-1 is complex and treatment of pwCF with GH has given conflicting results.^202^^,^^208^^,^^209^ No general recommendation for treatment of impaired growth with GH are given,^210^ which would be logical, if the low GH is only a marker of low IGF-1. A study in healthy infants showed that low IGF-1 was correlated to low LA concentration in serum phospholipids.^211^ In animal models of EFAD low IGF-1 and growth normalized, when the animal received standard diet normalizing the fatty acid profile.^212^^,^^213^ It would thus be of interest to examine prospectively any correlation between LA concentration, IGF-1 levels, and growth in pwCF.

Another factor related to poor growth is increased energy demand. One suggested cause to high energy demand is an *increased resting energy expenditure* (REE), a high metabolic turn-over related to the basic CF defect.^214^^,^^215^^,^^216^^,^^217^^,^^218^ It has been shown that severe CF phenotype, e.g., homozygotic for Phe508del, is associated with an increased resting respiratory rate,^215^^,^^218^^,^^219^^,^^220^ not always associated to pulmonary function, but to the pancreatic status.^221^ In rats with EFAD an increased basal respiration has been reported and related to growth retardation.^222^ The molar ratio of PC/phosphatidyl ethanolamine (PE) has been correlated with oxidative capacity and energy production in hepatic mitochondria,^223^ and the phospholipids of the membranes are crucial for the mitochondrial function.^224^ Independent of the plasma membrane potential, the mitochondrial membrane potential was decreased in CF airway cells non-related to chloride channel activity but to changed mitochondrial morphology.^225^ It has been suggested that when mitochondrial activity in CF respiratory cells is below a certain energy threshold,^223^ mitochondria dictate the conditions of the cells and this may reduce the disposition of the cells to infection related to reduced oxidative stress and altered glucose-related metabolic pathways.^226^ An increasing interest in the *mitochondrial dysfunction* in CF cells^227^^,^^228^^,^^229^ might possibly elucidate if the high REE is related to the mitochondrial dysfunction and membrane fatty acid configuration in CF.^230^^,^^231^ Cardiolipin (CL) is a major and important phospholipid in mitochondrial inner membranes required for optimal activity of several mitochondrial carrier proteins, and especially tetralinoleoyl-CL is important for optimal activity of the ADP/ATP carrier.^232^ In liver tissue from pwCF cardiolipin was decreased compared to controls (Strandvik et al. to be published).

### Pancreatic function

*Exocrine pancreatic insufficiency* develops early in about 85% of pwCF and is sometimes present at birth. It has been related to the impaired secretin response and the decreased bicarbonate secretion reducing the intestinal pH, described long before CFTR was identified.^233^ Interestingly, the impaired pancreas function in a newborn was reversed during Intralipid supplementation,^175^ but the results could not be replicated although Intralipid resulted in better growth.^176^ PwCF with more expressed LA deficiency, seem to have CFTR variants associated with more severe phenotypes (classes I-III) including growth impairment and pancreatic insufficiency.^57^ The pancreatic acini and ducts are obliterated by secretions in CF animal models, and it is not known if the impairment of the pancreatic secretion is initially due to a blockage in secretory ducts.^234^ In newborn CF pigs, which show EFAD already at birth,^126^ the acinar function is impaired with lower pH and impaired enzyme expression.^235^ Also the CF ferrets^126^ show EFAD at birth and have dilatations of acini gradually developing both endocrine and exocrine pancreatic insufficiency.^236^ In rats with EFAD, the amylase secretion was impaired and although responding to carbachol stimulation, it was not catching up to control levels.^237^ AA has been shown to be involved in the carbachol stimulated amylase secretion,^238^ which might be provided by its products, prostaglandins, as suggested in studies with inhibitors.^239^ In pancreatic acinar cells, LA much more than AA, stimulated basal amylase secretion by activating protein kinase C in the presence of Ca^2+^.^240^ The complex interaction of *n*-6 fatty acids in enzyme secretion needs further studies to find out if and how they are contributing to the pancreatic pathophysiology in CF.^241^^,^^242^

The *endocrine pancreatic function*, the insulin secretion, seems to be more impaired in pwCF with low LA concentrations (Hjelte, Strandvik et al., unpublished observation). Especially the first rapid increase of insulin was more impaired in pwCF with LA deficiency compared both to pwCF with normal LA concentrations and to healthy age-matched controls. An inverse correlation was found between plasma LA concentration and HbA1c.^243^ The increased prevalence of diabetes with age in pwCF suggests a gradual decline in insulin production and/or reduced insulin sensitivity,^244^ which is preceded by impaired glucose tolerance for years.^245^ In this context the report that high LA concentrations were protective for type 2 diabetes mellitus is of interest.^246^ The development of CF related diabetes mellitus (CFRD) is usually associated with clinical deterioration with decrease in weight and pulmonary function.^247^ In mice with the Phe508del mutations, the impairment in insulin secretion develops during the first 12 weeks of life together with impaired growth and reduced β-cell function.^248^ It is not clear if the endocrine impairment is independent of or to what extent it is related to the progression of pancreatic exocrine morphological deformation. In transgenic CF pigs the insulin homeostasis improved, when the pancreatic exocrine atrophy was completed, suggesting that the two processes were at least partly independent.^249^ In cells from human Langerhan’s isles AA was necessary for the glucose-stimulated insulin secretion.^250^ Humans with type2 diabetes and animals with alloxan-induced diabetes had low long chain fatty acid concentrations and prior administration of AA, EPA and DHA to the animals could protect them from induced diabetes.^251^ The effect of AA was not mediated by its prostanoid metabolites but by lipoxin LXA_4_, which production is impaired in CF.^252^ In this context it is of interest that one study showed that DHA supplementation increased LXA_4_ by probable switching the balance between 5-LOX and 15-LOX enzymes in the metabolism of AA.^253^ The role in CF of the balance between LA and AA, as well as that to DHA, and the possible influence on the gradual development of diabetes waits further studies of importance for the possibility of protection.

### Gastrointestinal function

The well-known impairment in duodenal *bicarbonate secretion* is not seen in the stomach of pwCF,^254^ probably because the gastric bicarbonate secretion is not dependent on CFTR, but on prostaglandins,^255^^,^^256^ which syntheses are generally increased in CF.^177^^,^^257^^,^^258^ This might explain the relatively good effect of modern pancreatic enzyme therapy without obvious benefit by bicarbonate supplementation.^259^ The bicarbonate secretion in duodenum and in the pancreatic ducts is stimulated by secretin, related to a functional CFTR, and is thus impaired in most pwCF.^233^

PwCF have increased *intestinal permeability,*^260^ which correlates to the clinically more severe phenotype Phe508del.^261^ Membrane phospholipid layers are important for the intestinal permeability and function^45^ and EFAD relates to both functional and morphological abnormalities.^262^^,^^263^^,^^264^ Ileum and colon in mice seem to be especially sensitive for EFAD with a 5-fold decrease of LA concentration in the mucosa compared to controls and without a similar decrease in blood.^85^ In a study of EFAD in rats, *morphological* changes were only noticed in ileum and not in jejunum,^263^ which together with the results in mice with EFAD,^85^ suggest that ileum is more sensitive to a deficiency state than the rest of the small intestine. Impaired active reabsorption of bile acids in ileum has been documented in both pwCF and animal models.^265^ Morphological changes and lipid abnormalities have been documented in ileum of CF mice.^266^ Nitric oxide synthase (NOS2) was reduced in ileum of CF mice in concordance with studies of human trachea, where loss of CFTR reduced NOS2messenger RNA expression and reduced overall NO production.^267^ Low NO may be related to abnormal lipids and bacterial growth also in the intestine, which has not been shown, but can be indicated by the studies of De Lisle,^268^ see below. In CFTR-KO mice, lipid mapping with cluster time of flight secondary-ion mass spectrometry (TOF-SIMS) imaging of the colon mucosa and lamina propria, showed low LA concentrations in the epithelial border, and principal component analyses showed marked differences between CF mice and wild type (WT) mice.^269^ Morphological abnormalities in the intestine of patients with CF are mainly described in ultramicroscopical studies,^270^ but increase of mucus producing cells are reported also in studies using light microscopy, including organoids.^271^ Occasionally it has been shown an improvement in pancreatic function by ETI,^272^ but improvement in general status and body weight suggest higher improvement than verified. Any effect on absorption by ETI treatment have not been studied, but a slight increase in serum levels of vitamin A and D, but not of vitamin E, was reported, although the variation was very large.^68^ Modern technology opens further possibility to study abnormalities of the lipid structure in relation to physiological disturbances in intact tissues and it would be of importance to further investigate the impact of the lipid abnormalities in the CF intestine in relation to physiology. It has been shown that newborns with meconium ileus often are more LA deficient than pwCF without this complication.^120^ The fact that ENaC is mainly expressed in colon^179^^,^^273^ implies its tentative importance for the development of meconium ileus and the comparative disease in the adult pwCF, the distal intestinal obstruction syndrome (DIOS).

PwCF have also been described to have *inflammation of the intestinal mucosa,*^274^ sometimes even referred to as colitis resembling Crohn's disease.^275^ Some studies found differences indicating that the inflammation in the CF intestine is different from that observed in inflammatory bowel disease, with higher fecal calprotectin and impaired growth.^88^^,^^276^^,^^277^^,^^278^^,^^279^ In a small study in pwCF with liver disease 4/11 reduced fecal calprotectin during 6 months of ETI treatment and changed the microbiota, but the study did not give any data of feeding and antibiotic treatment during the study period.^280^ In the intestine of transgenic CF mice there was a marked change in phospholipid and eicosanoid metabolism with high activity of COX 2 and its inflammatory product, PGE_2_.^268^ This might be related to high liberation of AA in the intestine, and might have relevance for the increased mucus production, similar to what has been shown in the airways, where high activity of PLA_2_ was associated with increase of mucins.^281^ The lipid abnormalities together with the impaired secretion by a dysfunctional CFTR and ENaC^282^ might contribute to the mucus abnormality increasing the risk of *intussusception* in CF.

A *disturbed motility* has been discussed as a possible factor for the gastrointestinal dysfunction and was shown for the circular muscle in CF mice.^283^ This was not verified in pwCF during fasting conditions, where differences could neither be seen in the longitudinal nor in the circular muscle contractions compared to non-CF controls.^254^ *Dysbiosis* has been verified in several studies and present also in comparisons with the influence of frequent use of antibiotics.^284^^,^^285^ It has been suggested that there is a gut-lung axis implicating that the disturbed intestinal milieu transforms cytokines and others to influence the lungs.^286^ Maybe surprisingly, no author has suggested that trends in these two organ systems may be related to the general lipid status, which might interfere with mucosal permeability, immunology, bacterial growth, and metabolism. Studies are warranted.

### Liver and biliary system

In CF both the liver and biliary tract are involved, and the pathophysiology is not fully understood, since CFTR is not shown to be expressed in the hepatocytes.^287^^,^^288^ On the other hand it has been suggested that small amounts of CFTR may be localized in the intracellular compartments with functions not clearly documented.^289^ Identification with antibodies may give cross reactivity but combined data do not support an expression in the hepatocytes*. Steatosis* is the most common finding and has sometimes been described as excessive,^290^ but mostly as a more or less distributed finding in the microscopical analyses of liver biopsies and in imaging investigations.^291^ Steatosis was found to be inversely associated to the LA concentration in plasma phospholipids,^291^ and CF related liver disease (CFRLD) was associated with lower DHA concentrations.^119^^,^^130^^,^^131^ Long-term studies in CF mice supplemented with DHA showed less periportal inflammation compared to a control group^146^ and some authors suggest that the ratio between AA/DHA in blood cell tissues is related to liver disease in CF and that the disturbed PUFA concentrations might contribute to the unexplained liver affection in many of the pwCF.^130^^,^^131^^,^^146^^,^^291^^,^^292^ Interestingly a long-term supplementation with Intralipid for 3 years showed less steatosis compared with untreated CF controls.^293^ Steatosis has been suggested to increase the risk of cirrhosis, and periportal fibrosis is common in pwCF and may in 5–10% result in a multinodular cirrhosis.^291^^,^^294^ The Swedish policy to supply LA,^291^^,^^295^ might therefore be a factor contributing to low prevalence of cirrhosis and portal hypertension in Swedish pwCF, shown in comparison with other centers in an international multicenter study comprising more than 1500 pwCF in 11 CF centers in Europe and Australia.^296^

Morphological signs of *inflammation* in the liver are relatively rare but a vascular inflammatory component has been discussed as a dominant feature in a smaller number of pwCF, who rapidly developed portal hypertension without cirrhosis.^297^^,^^298^ It would be of interest to study that group of pwCF in relation to lipids since endothelial cells are interactive with blood cells regarding cytokines and eicosanoids.^299^^,^^300^^,^^301^

The disturbance in *bile acid metabolism* and *biliary tract abnormalities* can be expected since CFTR is expressed in the cholangiocytes, including the gallbladder epithelium. Clinically, disturbed function and anatomy of the gallbladder, such as an enlargement of the latter (less than 10% of pwCF), or a non-visible micro gallbladder (>30%), are the most common findings. *Cholangiopathies,* visually similar to primary sclerosing cholangitis are not uncommon^302^ increasing in prevalence with age.^303^ This has been suggested to be linked to the abnormal CFTR function with sticky secretion, but in electron microscopical analyses of liver biopsies from pwCF the small cholangial ducts were not plugged although biochemically a cholestatic pattern was common.^304^^,^^305^ It is of interest that CFTR variants are frequently found in primary sclerosing cholangitis.^306^ Malignancy in the biliary system is not unusual in CF, but the risk of colon malignany is greater.^307^^,^^308^

It has been reported that pwCF have an increased fecal bile acid excretion,^309^ defective bile acid absorption and thus a low bile acid pool size.^310^ However, this was not confirmed in a study of Swedish pwCF in good clinical condition,^311^ and the absorption seems very good both after individual bile acid supplementation and after a test meal (Strandvik et al., unpublished observation). It cannot be excluded that the varying results are related to the clinical condition of the pwCF. The bile acid pattern in serum, urine and duodenum showed that patients with CF have increased primary bile acids, cholic and chenodeoxycholic acids; the latter increase mainly related to CFRLD.^312^^,^^313^ The secondary bile acids, known to be more toxic than the primary ones, are sometimes decreased in CF.^309^^,^^312^ Interestingly a recent report found increase of deoxycholic acid, more pronounced in CFRLD without clinical cirrhosis.^314^ If differences are associated with treatment strategies, including influence from the intestinal microbiome, have not been reported. Interestingly, long-term supplementation of LA resulted in some improvement of the plasma bile acid profile.^293^ The complex fat metabolism in the liver restrict any suggestions of relation to the impaired metabolism and need more focused studies.

### Kidney function

PwCF do not usually present clinical impairment in renal function, except after long-term treatment with toxic drugs, like aminoglycoside antibiotics or after transplantation with extensive immunosuppressive therapy.^315^ However, the kidneys are involved in CF,^316^ and pwCF have a delayed renal *acidifying capacity*, indicating a reduced bicarbonate threshold, which was interpreted as a proximal tubular dysfunction.^317^^,^^318^ This is supported by CFTR being mainly expressed in the proximal tubules and in the collecting ducts, which were shown to have an impaired secretin response long before the gene was discovered.^233^

*Renal sodium excretion* is impaired in infants with CF despite supplementation^182^ and is also lower in children with CF than in controls.^317^^,^^319^ This low excretion was not improved by 10 days of high sodium intake or after intravenously loading with high doses.^317^ Investigations with ^24^Na did not indicate sodium depletion.^320^ Three years of LA supplementation to children with CF resulted in an improvement and even normalization in the renal sodium excretion,^318^ but since the study was performed before the gene was discovered, differences in relation to mutation were not investigated. The low sodium excretion has also been correlated to poor growth and interpreted as low sodium status despite normal plasma levels of sodium.^319^^,^^321^ ENaC is present in the renal tubules,^322^^,^^323^ and similar hyperactivity as in the airways, might explain the decreased sodium excretion.^324^^,^^325^ The LXR might also influence ENaC mediated sodium reabsorption in collecting duct cells.^326^ The results, including those in sweat (see above), indicate that the intramembranous ENaC activity directly or indirectly was influenced by LA supplementation, supported by studies relating ENaC activity to rafts.^323^

PwCF have an increased *glomerular filtration rate* which was normalized after fatty acid supplementation.^318^^,^^327^^,^^328^ It is well-known that pwCF need high doses of antibiotics^329^ partly due to an increased non-renal clearance^330^ and partly due to increased glomerulus filtration rate.^331^ After LA supplementation the glomerulus clearance normalized.^318^ In rats with EFAD, glomerulus clearance was increased.^332^

In a dietary study of tissue incorporation of EFA in rats, the LA levels were 18-fold higher in kidney phospholipids than that of alpha-linolenic acid (ALA, 18:3*n*-3), both provided in similar amounts.^333^ Oxylipins from LA were highly concentrated in the kidney,^333^^,^^334^ but not related to the AA concentration or the corresponding AA products, suggesting that the effect was not eicosanoid related. In a study of EFAD rats, labeled LA was highly incorporated in the kidney, especially in PE but not in phosphatidylinositol (PI).^335^ In another experimental study of mice with EFAD, AA was well preserved in PC, PE and phosphatidyl serine (PS) in the kidney compared to the liver, but not in PI.^336^ Nothing is known about the effect in CF, but tentatively the transmembrane proteins CFTR and ENaC may be influenced directly or indirectly^326^ by LA deficiency changing membrane lipid pathophysiology affecting the described disturbances as indicated by the effect of LA supplementation. PGE_2_ and PGF_2α_, which are increased in CF, enhance the ENaC channel open probability.^337^

### Airway function

The problem for many years has been the controversial concept of what is the primary origin of the airway problems - is it inflammation *per se* or the infections causing inflammation (for review see^137^). Increasing amount of data indicate that the *inflammation starts before the infections*^338^^,^^339^ supporting an abnormality in the inflammatory homeostasis, beginning already *in utero.*^340^ The inflammation might be related to the increase of AA,^341^^,^^342^ which is rate-limiting for the synthesis of eicosanoids, most of which are pro-inflammatory and found in high amounts in the airways, but also in urine indicating a general high production.^177^^,^^257^^,^^258^ High AA concentrations are also related to *increase of MUC5A,* important for the pathological mucus in CF,^281^ and to low exhaled NO, also in non-pseudomonas infected pwCF.^343^ The low exhaled NO was inversely correlated to EFAD index.^344^ Infection will add to a primary inflammation, contributing to the severity of the pulmonary disease in CF. Such interpretation is supported by studies reporting improvement of the lipid abnormality in serum after lung transplantation, not in terms of the disturbed balance, but in the total absolute amount of LA.^345^ Significant correlations have been reported between pulmonary function and the LA status of pwCF.^116^^,^^119^^,^^243^^,^^346^ Interestingly also in a chicken model pulmonary pathology was induced by EFAD.^347^ In Sweden with decades of LA supplementation in the two largest CF centers, the lung function has been higher than in the other Nordic centers despite lower antibiotic use.^295^^,^^348^^,^^349^ The defective macrophage function, of importance for the bacterial clearance in the airways, is dependent on several factors and modulators have contradictory results.^350^ High PGE_2_ in pwCF inhibited phagocytosis of inhaled diesel exhaust particles.^351^ ETI had some influence on the phagocytosis in pwCF, but did not influence the inflammatory markers.^352^ The cell membrane lipid configuration would be interesting to investigate in this context.

### Bone mineral density

Osteoporosis and fractures are reported as a common finding in adults with CF,^353^ but are rare in the Swedish CF population.^354^ PwCF with normal growth have decreased bone mineralization but normal bone growth was found in a longitudinal study of the Swedish pwCF, who regularly received LA supplementation.^355^^,^^356^ The bone mineral content was differently associated to the fatty acid profile in children and adult pwCF,^357^^,^^358^ which can be referred to different influence of fatty acids during bone modeling in childhood and bone remodeling in adults.^359^ Animal studies have shown that fatty acids are important for bone growth.^212^^,^^213^^,^^360^^,^^361^^,^^362^ The role of PGE_2_ in bone modeling is depending on its concentration and there are indications in animal experiments that IGF-1, which is low in CF, is important for mineralization of trabecular bone.^363^^,^^364^ Studies in healthy children indicate that both LA and DHA are important for bone mineral density.^365^^,^^366^ Longitudinal studies are warranted to evaluate associations between bone modeling and lipid metabolism in CF.

## Clinical effects of fatty acid supplementation

After 1975, when the pancreatic function in a newborn infant with CF temporarily recovered after Intralipid administration,^175^ several trials with LA, or high energy diets including LA, have been performed, a few for one year or longer.^80^^,^^174^^,^^346^ None showed influence on pancreatic function, but influence on growth, also in supplementation for only some months.^180^^,^^198^ High energy intake alone did not have the same effect.^180^^,^^182^^,^^367^^,^^368^ Pulmonary function was also influenced by LA status but not by *n*-3 fatty acid supplementation.^116^^,^^243^^,^^369^

In a long-term controlled small study performed before the gene was identified, administration of Intralipid biweekly over 2 of 3 years,^174^ showed improvements in renal and liver parameters compared to the control pwCF not receiving LA supplementation.^293^^,^^318^ The dose was relatively low, which was illustrated by a very slow improvement in plasma fatty acid status. Another study showed increased LA in several tissues after one year of LA supplementation compared to non-supplemented pwCF.^80^ Many clinical studies have shown associations between low LA and clinical symptoms.^116^^,^^119^^,^^131^^,^^243^^,^^291^^,^^346^^,^^370^ Animal studies of LA deficiency have shown similar clinical symptomatology as found in pwCF.^237^^,^^332^^,^^347^ It has to be noticed that most studies have given too low substitution or for too short time. In one study giving very high amount of LA (13 g per serving) the effect in some children was remarkable.^187^ In relation to all available data about low LA, reported for more than 60 years, it is surprising that nutritional guidelines usually not mention the fatty acids. One reason might be that the fatty acids are not analyzed and thereby not considered. Thus, there are no general recommendations for LA supplementation.^371^^,^^372^

It is well known that low LA concentrations sometimes are associated with high AA concentration, while supplementation with LA decreases the AA concentration,^138^^,^^139^^,^^140^^,^^141^^,^^373^ and importantly - without increasing inflammation.^139^ The general inverse relation in plasma phospholipids between AA and LA is also documented in pwCF.^137^ Based on results in cell cultures using sense and antisense CF cells, warnings have been expressed to supply LA in CF,^374^^,^^375^ which might explain the reluctance to further explore this simple treatment strategy. It is of interest that the eicosanoid excretion was influenced by LA supplementation, supporting the inverse relation between LA and AA.^177^ Obviously also in Sweden, where LA has been supplied to most pwCF at the two largest centers for several decades, the good clinical results were thought to depend on the general pulmonary treatment strategy, especially the antibiotic schedules.^376^^,^^377^^,^^378^ It is difficult to compare small centers, which of course have small differences in general treatments, but what really stands out when comparing the Nordic centers was the lipid supplementation policy (only applicated in the two biggest centers in Sweden), which was associated with less need of antibiotics and still good lung function.^295^^,^^348^^,^^349^ This observation motivates a double-blind randomized study to evaluate if regular LA supplementation should be offered to the pwCF as an adjuvant therapy (ClinicalTrials.gov ID#NCT04531410).

The cause of the low DHA is not clear, but the variation in concentration in different pwCF and models suggests that this might not be a primary disturbance. Some improvement of DHA by LA supplementation has been shown supporting it might be secondary to the lipid dysfunction^125^ associated with a high phospholipid turn-over and a supply of PC via the alternative pathway from PE, rich in DHA.^108^ This is further supported by the general lack of clinical improvement by *n*-3 supplementation studies.^147^^,^^148^^,^^379^^,^^380^^,^^381^^,^^382^ One longitudinal study showed positive association between *n*-3 fatty acids and lung function,^383^ and one study showed that high ratio between the DHA-derived resolvin D1 and IL-8 levels in sputum was associated with better lung function.^384^ However, another study with regular intravenous supplementation of omega-3 for 3 months showed negative impact on the included pwCF.^385^ One *n*-3 supplementation study showed marked clinical improvement by a combination with γ-linolenic acid, the fatty acid derived from LA in the transformation to AA.^75^^,^^386^ The *n*-3 fatty acids are important as substrate for SPM.^387^ Thus, if not possible to balance a high eicosanoid production by decreasing the AA release, the counter action of *n*-3 providing SPM might theoretically balance the inflammation, which was indicated in cell cultures.^388^ That was also supported in a community-based study of non-CF adults, where the total *n*-3 fatty acids were independently associated with lower levels of proinflammatory markers and higher levels of anti-inflammatory markers.^389^ Interesting observations were reported in studies of mice^253^ and infants,^390^ respectively, where DHA supplementation highly amplified 15-LOX increasing LXA_4_ in plasma compared to controls, and thereby influencing inflammation. It was also possible to improve LA by DHA supplementation in cell culture studies with sense-antisense cells.^391^ Current evidence is insufficient to routinely recommend supplementation of omega-3 fatty acids to people with CF, although theoretically it may hamper inflammation.

The potentiator Ivacaftor given to pwCF with the G551D mutation decreased AA and also showed a trend to less prostanoid excretion,^392^ but hitherto studies in pwCF have not shown influence on the classical plasma fatty acid pattern by the new modulators,^69^^,^^71^ despite that several studies have shown a general influence on PC and ceramides.^40^^,^^41^^,^^42^^,^^43^^,^^44^^,^^155^^,^^393^

## Possible mechanisms of the fatty acid abnormalities

The mechanism underlying CFTR and the fatty acid abnormalities is not known and suggestions have previously been discussed.^108^ CFTR seems to be inhibited by AA like other chloride transporting epithelial channels,^394^^,^^395^ and the mechanism has been suggested to be an electrostatic interaction with amino acids in blocking the entrance in the CFTR molecule.^396^ This might only be relevant for the classes of mutations with disturbed gating. The multi-complexity of disturbed functions of the more than 2200 variants of CFTR indicates additional mechanisms. The recent information that the new modulators influence the phospholipids and ceramides opens for new aspects and interest in the possible interaction between CFTR and lipid metabolism. Abu-Arish et al.^61^^,^^62^ have suggested that there might be two populations of CFTR related to membrane cholesterol enrichment influencing the micro-domains and raft platforms. These aggregations were regulated by secretagogues like vasoactive intestinal peptide and carbachol, which increased the CFTR aggregation into clusters building ceramide-rich platforms.

It is well known that lipids modulate ion channel activity, either by direct interaction with the channel structure or by modulating the physio-chemical properties of the cellular membrane.^50^^,^^397^ The importance of protein-lipid interaction for normal activity of channels and other membrane bound proteins are well established.^398^^,^^399^^,^^400^^,^^401^ The fatty acid composition influences enzyme activity, receptor affinity, transport capacity, and permeability,^75^ as illustrated in the activity of Ca^++^-ATPas being related to the chain length of fatty acids,^76^ and the function of aquaporins dependent on binding to phospholipids.^77^ One factor might be that different mutations of CFTR make the stereo-interaction with the lipids in membranes incomplete thereby disturbing the membrane associated transports or the activity of the channel in the plasma membrane.^75^^,^^76^^,^^77^^,^^402^^,^^403^^,^^404^ Support for such mechanism in CF was presented by Eidelman et al.^405^ showing that the NBF-1 domain of CFTR interacted in membranes with PS rather than PC, but that the Phe508del mutation lost the ability to discriminate between PS and PC. They showed that non-charged analogous to PC could increase the CFTR expression, indicating that phospholipid chaperones might be therapeutic tools. This work links clinical PC abnormalities with the CFTR dysfunction. In this context it is of interest that PS was shown to thermally stabilize CFTR by stimulating the ATP hydrolysis function of purified CFTR. Regulatory lipid interactions may differently regulate ion channel functions in different organelles,^406^ explaining or contributing to different abnormalities in different tissues (for overview see Figure 1).

It was suggested more than 30 years ago that the LA deficiency was not due to malabsorption but related to an *increased release of AA* with an increased compensatory transformation of LA, resulting in low LA concentrations.^342^^,^^407^ The AA release from membranes was not inhibited by dexamethasone in cells from pwCF compared to healthy controls, indicating an increased activity of cPLA_2_, the major AA releasing enzyme, which was confirmed by others in different cell systems.^281^^,^^408^^,^^409^^,^^410^ A high turn-over demands upregulation of the enzymes involved in the fatty acid transformation, which was documented and suggested as the primary defect.^411^ The upregulation of the desaturases and elongases was related to *increased activity of cAMP-activated protein kinase (AMP/APK),*^412^ which might be secondary to infection/inflammation and thus further increasing the transfer of LA to AA.^413^

The increased AA release was suggested being the result of an *impairment in the regulation of cPLA*_*2*_ *by annexin 1* (lipomodulin) or the necessary phosphorylation for its action on cPLA_2._^342^ (Figure 1). This hypothesis has been supported in reports that annexin 1 is decreased in CF cells and that inhibition of annexin 1 stimulates AA release^414^ and in cell systems also attenuate glucocorticoid functions.^415^ In the annexin-1 KO mice macrophage functions are impaired with increased COX-2 and PGE_2_ release with an exaggerated response to inflammation.^416^^,^^417^^,^^418^ Annexin 1 was shown to colocalize with PS for clearance of apoptotic cells,^419^ illustrating the importance of phospholipids in cell functions. The activity of annexin 1 is linked to S100, low molecular weight proteins of calcium-binding family, which also have been found involved in the cAMP/PKA signaling.^420^ Borthwick et al.^421^ showed that the annexin 2-S100A10 complex with CFTR regulated the channel function by AMP/PKA and showed this action was defective in Phe508del cells. In neutrophils, LA and its fatty acid derivatives are ligands to fatty acid binding complex with S100 proteins.^422^ It is of interest that strains of CF mice with different expression of the S100 proteins had different correlations to inflammatory lung disease and increase of neutrophils.^423^ In a recent study of bronchoalveolar lavage fluid of young pwCF 1–5 years of age, a shift was shown from LA to AA derivatives associated with neutrophil influx and progression of lung damage,^72^ supporting studies in annexin 1-deficient neutrophils showing a greater extent of leukocyte transmigration resulting in a prolonged and exacerbated inflammatory response.^424^ However, the interaction between CFTR and annexin or its complex with S100 is not known. Of interest for functional studies might be that the eight annexins have 37–47% homology with CFTR.^425^

If AA release is primary to LA deficiency in CF, a blockage of the AA release would have therapeutic implication, as reviewed recently.^426^ If it can be confirmed that LA supplementation in CF would be a way to naturally decrease the availability of AA,^138^^,^^139^^,^^140^^,^^141^ thereby circumventing the lacking CFTR/annexin/S100 inhibition of PLA_2_, that would be a tentative way to decrease the inflammation. The good results in Sweden by supplementation of LA for decades before the modulator therapy was available might support such suggestion.^295^^,^^348^^,^^349^^,^^378^

Activation of AMP/APK may also interfere with ENaC to decrease sodium absorption and reduce inflammation.^427^^,^^428^ An *upregulation of ENaC* has been documented in many experimental studies, and considered to contribute to the impaired secretion.^429^ Interestingly, overexpression of one trimer of the ENaC molecule results in CF like clinical disease.^5^ It has been shown that CFTR influences ENaC activity, but the mechanism is unclear. Some authors have suggested a close localization between the two channels in the membranes,^430^^,^^431^^,^^432^ but that was not supported in a study on alveolar cells.^433^ Whatever, the mechanism, it is clinically clear that LA administration influences sodium excretion both in sweat and kidneys.^324^ Since both CFTR and ENaC are active in membranes, it might be speculated that lipid membrane configuration interferes with their functions. It has been shown in cell studies that AA, like its product by cytochrome P450 epoxygenase, the 11,12-EET, significantly reduced the ENaC open probability, but that it was significantly enhanced by PGE_2_ and PGF_2α,_^337^ suggesting importance of the AA metabolism for the symptoms. Increased ENaC activity can have different explanations and Na^+^-K^+^-ATPas activity was coordinated with the ENaC activity in studies in renal collecting ducts.^434^ It is therefore of interest that the Na^+^-K^+^-ATPas activity in erythrocytes was activated by LA supplementation.^435^ It has been suggested that an inhibition of ENaC would improve the symptoms in CF and modulator development for ENaC are in progress.^436^ If the sodium transport can be improved by supplying LA it would be an inexpensive therapeutic additive.

Another factor of interest for the pathophysiology is the similarity between *LXRβ* KO- mice and clinical CF symptomatology.^3^^,^^164^ LXRβ interferes with CFTR,^4^^,^^437^^,^^438^ cholesterol, and fatty acid metabolism.^165^ LXRs influence immune response and inflammation^439^^,^^440^ and are important regulators of inflammatory gene expressions, and can blunt COX-2 and iNOS genes and various chemokines in response to TNF-α and IL1-β.^439^^,^^440^^,^^441^ LXRαβ−/− mice have shown increased susceptibility to infection associated with defective macrophage function,^442^ which is of interest since LXRs interfere with AA metabolism and eicosanoid secretion in macrophages.^443^ An important mediator of LXR effects is the modulation of PUFA in PC by influencing lysophosphatidylcholine acyltransferase 3, which preferentially synthesizes PC containing AA and LA at the sn-2 position.^107^ The different impact on or result of defective annexins and LXR on the fatty acid metabolism and CFTR are challenges for understanding the pathophysiology and symptoms in CF.

The influence of fatty acids on *gene expression* must also be considered.^444^ An increasing number of reports put attention to overlapping influences of CFTR variants, and different effects of therapy suggesting that the classification of six groups of mutation related dysfunctions probably need to be modified.^445^^,^^446^ This indicates that personalized therapy will be more important than only a broad classification of the CFTR variants. In this context it would be of interest to investigate the fatty acid status of the pwCF related to different effects on/of CFTR variants, since the fatty acid composition differs in cell organelles and plasma membranes which would be an explanation to different levels of dysfunction or blocking.^403^^,^^447^

Membrane lipid therapy has been suggested as possible treatment for many pathological conditions.^448^ The clinical associations between dysfunction of LA, AA, and DHA and the CF symptomatology related to the membrane proteins CFTR and ENaC, as well as to the nuclear receptor LXRβ, supported by different observational, experimental, and animal studies as outlined in this review, suggest that further studies on the lipid abnormalities are relevant, and may open for new and even cheap concepts for additional treatment.

## Conclusion and prospects

The lipid abnormalities in CF are present at birth and develop further, more in organs than in serum, if not compensated by supplementation of LA by high dietary intake and/or intravenous supplementation. As components of membranes, fatty acids influence many metabolic functions and the fatty acid products, like the lipid mediators, are of strong importance for both metabolic and inflammatory characteristics of CF. The transmembrane localization of both CFTR and ENaC explains interaction with lipids. The role of annexin/p11 controlling the AA release might be a key factor in the pathophysiology, explaining high AA and compensatory low LA. Despite the accumulating evidence from both observational and experimental studies regarding the tentative relation between symptoms in CF and lipid abnormalities and the potential clinical benefits of fatty acid supplementation, lipids are not routinely analyzed, and no recommendations are provided in clinical guidelines. Confirming the possible additive or synergistic effects of fatty acid supplementation to CFTR modulating therapy would open for inexpensive and important adjuvant therapy and might even have an influence in those pwCF not eligible for the available modulator therapy.

## Author contributions

B.S. designed the original draft, but all authors contributed with literature search and conceptualization of the manuscript. B.S. reviewed and edited the manuscript, which was approved by all authors.

## Declaration of interests

J.W. reports personal fees and non-financial support from Biocodex, BGP Products, Chiesi, Hipp, Humana, Mead Johnson Nutrition, Merck Sharp & Dohme, Nestle, Norsa Pharma, Nutricia, Roche, Sequoia Pharmaceuticals, and Vitis Pharma, outside the submitted work, and grants, personal fees and non-financial support from Nutricia Research Foundation Poland, all outside the submitted work.

None of the other authors declare any conflicts of interest. Financial support for discussion meetings was received from European Society of Pediatric Gastroenterology, Hepatology and Nutrition and from the Swedish Cystic Fibrosis Association. No competing interest are reported by the authors.

## References

[1] Rubin B.K. (2018). Unmet needs in cystic fibrosis. Expet Opin. Biol. Ther..

[2] West N.E., Flume P.A. (2018). Unmet needs in cystic fibrosis: the next steps in improving outcomes. Expet Rev. Respir. Med..

[3] Gabbi C., Warner M., Gustafsson J.A. (2009). Minireview: liver X receptor beta: emerging roles in physiology and diseases. Mol. Endocrinol..

[4] Sweed N., Kim H.J., Hultenby K., Barros R., Parini P., Sancisi V., Strandvik B., Gabbi C. (2021). Liver X receptor β regulates bile volume and the expression of aquaporins and cystic fibrosis transmembrane conductance regulator in the gallbladder. Am. J. Physiol. Gastrointest. Liver Physiol..

[5] Mall M., Grubb B.R., Harkema J.R., O'Neal W.K., Boucher R.C. (2004). Increased airway epithelial Na+ absorption produces cystic fibrosis-like lung disease in mice. Nat. Med..

[6] Bierlaagh M.C., Muilwijk D., Beekman J.M., van der Ent C.K. (2021). A new era for people with cystic fibrosis. Eur. J. Pediatr..

[7] Saluzzo F., Riberi L., Messore B., Loré N.I., Esposito I., Bignamini E., De Rose V. (2022). CFTR Modulator Therapies: Potential Impact on Airway Infections in Cystic Fibrosis. Cells.

[8] Egan M.E. (2022). Non-Modulator Therapies: Developing a Therapy for Every Cystic Fibrosis Patient. Clin. Chest Med..

[9] Allen L., Allen L., Carr S.B., Davies G., Downey D., Egan M., Forton J.T., Gray R., Haworth C., Horsley A. (2023). Future therapies for cystic fibrosis. Nat. Commun..

[10] Stylemans D., Darquenne C., Schuermans D., Verbanck S., Vanderhelst E. (2022). Peripheral lung effect of elexacaftor/tezacaftor/ivacaftor in adult cystic fibrosis. J. Cyst. Fibros..

[11] Middleton P.G., Mall M.A., Dřevínek P., Lands L.C., McKone E.F., Polineni D., Ramsey B.W., Taylor-Cousar J.L., Tullis E., Vermeulen F. (2019). Elexacaftor-Tezacaftor-Ivacaftor for Cystic Fibrosis with a Single Phe508del Allele. N. Engl. J. Med..

[12] Jia S., Taylor-Cousar J.L. (2023). Cystic Fibrosis Modulator Therapies. Annu. Rev. Med..

[13] Foucaud P., Mercier J.C. (2023). CFTR pharmacological modulators: A great advance in cystic fibrosis management. Arch. Pediatr..

[14] Kapouni N., Moustaki M., Douros K., Loukou I. (2023). Efficacy and Safety of Elexacaftor-Tezacaftor-Ivacaftor in the Treatment of Cystic Fibrosis: A Systematic Review. Children.

[15] Thakur S., Ankita, Dash S., Verma R., Kaur C., Kumar R., Mazumder A., Singh G. (2024). Understanding CFTR Functionality: A Comprehensive Review of Tests and Modulator Therapy in Cystic Fibrosis. Cell Biochem. Biophys..

[16] Olivier M., Kavvalou A., Welsner M., Hirtz R., Straßburg S., Sutharsan S., Stehling F., Steindor M. (2023). Real-life impact of highly effective CFTR modulator therapy in children with cystic fibrosis. Front. Pharmacol..

[17] Bell S.C., Mall M.A., Gutierrez H., Macek M., Madge S., Davies J.C., Burgel P.R., Tullis E., Castaños C., Castellani C. (2020). The future of cystic fibrosis care: a global perspective. Lancet Respir. Med..

[18] King J.A., Nichols A.L., Bentley S., Carr S.B., Davies J.C. (2022). An Update on CFTR Modulators as New Therapies for Cystic Fibrosis. Paediatr. Drugs.

[19] Graeber S.Y., Mall M.A. (2023). The future of cystic fibrosis treatment: from disease mechanisms to novel therapeutic approaches. Lancet.

[20] Gillan J.L., Davidson D.J., Gray R.D. (2021). Targeting cystic fibrosis inflammation in the age of CFTR modulators: focus on macrophages. Eur. Respir. J..

[21] Roesch E.A., Nichols D.P., Chmiel J.F. (2018). Inflammation in cystic fibrosis: An update. Pediatr. Pulmonol..

[22] Ghigo A., Prono G., Riccardi E., De Rose V. (2021). Dysfunctional Inflammation in Cystic Fibrosis Airways: From Mechanisms to Novel Therapeutic Approaches. Int. J. Mol. Sci..

[23] McElvaney O.J., Wade P., Murphy M., Reeves E.P., McElvaney N.G. (2019). Targeting airway inflammation in cystic fibrosis. Expet Rev. Respir. Med..

[24] Perrem L., Ratjen F. (2023). Are we there yet? The ongoing journey of cystic fibrosis care. Lancet.

[25] Keown K., Brown R., Doherty D.F., Houston C., McKelvey M.C., Creane S., Linden D., McAuley D.F., Kidney J.C., Weldon S. (2020). Airway Inflammation and Host Responses in the Era of CFTR Modulators. Int. J. Mol. Sci..

[26] Griese M., Costa S., Linnemann R.W., Mall M.A., McKone E.F., Polineni D., Quon B.S., Ringshausen F.C., Taylor-Cousar J.L., Withers N.J. (2021). Safety and Efficacy of Elexacaftor/Tezacaftor/Ivacaftor for 24 Weeks or Longer in People with Cystic Fibrosis and One or More F508del Alleles: Interim Results of an Open-Label Phase 3 Clinical Trial. Am. J. Respir. Crit. Care Med..

[27] Graeber S.Y., Vitzthum C., Pallenberg S.T., Naehrlich L., Stahl M., Rohrbach A., Drescher M., Minso R., Ringshausen F.C., Rueckes-Nilges C. (2022). Effects of Elexacaftor/Tezacaftor/Ivacaftor Therapy on CFTR Function in Patients with Cystic Fibrosis and One or Two F508del Alleles. Am. J. Respir. Crit. Care Med..

[28] Mederos-Luis E., González-Pérez R., Poza-Guedes P., Álava-Cruz C., Matheu V., Sánchez-Machín I. (2022). Toxic epidermal necrolysis induced by cystic fibrosis transmembrane conductance regulator modulators. Contact Dermatitis.

[29] Salehi M., Iqbal M., Dube A., AlJoudeh A., Edenborough F. (2021). Delayed hepatic necrosis in a cystic fibrosis patient taking Elexacaftor/Tezacaftor/Ivacaftor (Kaftrio). Respir. Med. Case Rep..

[30] Tindell W., Su A., Oros S.M., Rayapati A.O., Rakesh G. (2020). Trikafta and Psychopathology in Cystic Fibrosis: A Case Report. Psychosomatics.

[31] Petersen M.C., Begnel L., Wallendorf M., Litvin M. (2022). Effect of elexacaftor-tezacaftor-ivacaftor on body weight and metabolic parameters in adults with cystic fibrosis. J. Cyst. Fibros..

[32] Despotes K.A., Ceppe A.S., Donaldson S.H. (2023). Alterations in lipids after initiation of highly effective modulators in people with cystic fibrosis. J. Cyst. Fibros..

[33] Heo S., Young D.C., Safirstein J., Bourque B., Antell M.H., Diloreto S., Rotolo S.M. (2022). Mental status changes during elexacaftor/tezacaftor/ivacaftor therapy. J. Cyst. Fibros..

[34] Spoletini G., Gillgrass L., Pollard K., Shaw N., Williams E., Etherington C., Clifton I.J., Peckham D.G. (2022). Dose adjustments of Elexacaftor/Tezacaftor/Ivacaftor in response to mental health side effects in adults with cystic fibrosis. J. Cyst. Fibros..

[35] Baroud E., Chaudhary N., Georgiopoulos A.M. (2023). Management of neuropsychiatric symptoms in adults treated with elexacaftor/tezacaftor/ivacaftor. Pediatr. Pulmonol..

[36] Arslan M., Chalmers S., Rentfrow K., Olson J.M., Dean V., Wylam M.E., Demirel N. (2023). Suicide attempts in adolescents with cystic fibrosis on Elexacaftor/Tezacaftor/Ivacaftor therapy. J. Cyst. Fibros..

[37] Bathgate C.J., Muther E., Georgiopoulos A.M., Smith B., Tillman L., Graziano S., Verkleij M., Lomas P., Quittner A. (2023). Positive and negative impacts of elexacaftor/tezacaftor/ivacaftor: Healthcare providers' observations across US centers. Pediatr. Pulmonol..

[38] Southern K.W., Addy C., Bell S.C., Bevan A., Borawska U., Brown C., Burgel P.R., Button B., Castellani C., Chansard A. (2024). Standards for the care of people with cystic fibrosis; establishing and maintaining health. J. Cyst. Fibros..

[39] O'Connor M.G., Seegmiller A. (2017). The effects of ivacaftor on CF fatty acid metabolism: An analysis from the GOAL study. J. Cyst. Fibros..

[40] Zardini Buzatto A., Abdel Jabar M., Nizami I., Dasouki M., Li L., Abdel Rahman A.M. (2021). Lipidome Alterations Induced by Cystic Fibrosis, CFTR Mutation, and Lung Function. J. Proteome Res..

[41] Despotes K.A., Donaldson S.H. (2022). Current state of CFTR modulators for treatment of Cystic Fibrosis. Curr. Opin. Pharmacol..

[42] Veltman M., De Sanctis J.B., Stolarczyk M., Klymiuk N., Bähr A., Brouwer R.W., Oole E., Shah J., Ozdian T., Liao J. (2021). CFTR Correctors and Antioxidants Partially Normalize Lipid Imbalance but not Abnormal Basal Inflammatory Cytokine Profile in CF Bronchial Epithelial Cells. Front. Physiol..

[43] Liessi N., Pedemonte N., Armirotti A., Braccia C. (2020). Proteomics and Metabolomics for Cystic Fibrosis Research. Int. J. Mol. Sci..

[44] Abu-Arish A., Pandžić E., Luo Y., Sato Y., Turner M.J., Wiseman P.W., Hanrahan J.W. (2022). Lipid-driven CFTR clustering is impaired in cystic fibrosis and restored by corrector drugs. J. Cell Sci..

[45] Hedger G., Sansom M.S.P. (2016). Lipid interaction sites on channels, transporters and receptors: Recent insights from molecular dynamics simulations. Biochim. Biophys. Acta.

[46] Hulbert A.J., Turner N., Storlien L.H., Else P.L. (2005). Dietary fats and membrane function: implications for metabolism and disease. Biol. Rev. Camb. Phil. Soc..

[47] Harayama T., Shimizu T. (2020). Roles of polyunsaturated fatty acids, from mediators to membranes. J. Lipid Res..

[48] Andersen O.S., Koeppe R.E. (2007). Bilayer thickness and membrane protein function: an energetic perspective. Annu. Rev. Biophys. Biomol. Struct..

[49] Phillips R., Ursell T., Wiggins P., Sens P. (2009). Emerging roles for lipids in shaping membrane-protein function. Nature.

[50] Poveda J.A., Marcela Giudici A., Lourdes Renart M., Morales A., González-Ros J.M. (2017). Towards understanding the molecular basis of ion channel modulation by lipids: Mechanistic models and current paradigms. Biochim. Biophys. Acta Biomembr..

[51] Bhura-Bandali F.N., Suh M., Man S.F., Clandinin M.T. (2000). The deltaF508 mutation in the cystic fibrosis transmembrane conductance regulator alters control of essential fatty acid utilization in epithelial cells. J. Nutr..

[52] Njoroge S.W., Seegmiller A.C., Katrangi W., Laposata M. (2011). Increased Δ5- and Δ6-desaturase, cyclooxygenase-2, and lipoxygenase-5 expression and activity are associated with fatty acid and eicosanoid changes in cystic fibrosis. Biochim. Biophys. Acta.

[53] Rogiers V., Dab I., Michotte Y., Vercruysse A., Crokaert R., Vis H.L. (1984). Abnormal fatty acid turnover in the phospholipids of the red blood cell membranes of cystic fibrosis patients (*in vitro* study). Pediatr. Res..

[54] Thomsen K.F., Laposata M., Njoroge S.W., Umunakwe O.C., Katrangi W., Seegmiller A.C. (2011). Increased elongase 6 and Δ9-desaturase activity are associated with n-7 and n-9 fatty acid changes in cystic fibrosis. Lipids.

[55] Ulane M.M., Butler J.D., Peri A., Miele L., Ulane R.E., Hubbard V.S. (1994). Cystic fibrosis and phosphatidylcholine biosynthesis. Clin. Chim. Acta.

[56] Guerrera I.C., Astarita G., Jais J.P., Sands D., Nowakowska A., Colas J., Sermet-Gaudelus I., Schuerenberg M., Piomelli D., Edelman A., Ollero M. (2009). A novel lipidomic strategy reveals plasma phospholipid signatures associated with respiratory disease severity in cystic fibrosis patients. PLoS One.

[57] Strandvik B., Gronowitz E., Enlund F., Martinsson T., Wahlström J. (2001). Essential fatty acid deficiency in relation to genotype in patients with cystic fibrosis. J. Pediatr..

[58] Hubbard V.S., Dunn G.D., di Sant'Agnese P.A. (1977). Abnormal fatty-acid composition of plasma-lipids in cystic fibrosis. A primary or a secondary defect?. Lancet.

[59] Galabert C., Filliat M., Chazalette J.P. (1978). Fatty-acid composition of serum-lecithins in cystic-fibrosis patients without steatorrhoea. Lancet.

[60] Liessi N., Pesce E., Braccia C., Bertozzi S.M., Giraudo A., Bandiera T., Pedemonte N., Armirotti A. (2020). Distinctive lipid signatures of bronchial epithelial cells associated with cystic fibrosis drugs, including Trikafta. JCI Insight.

[61] Abu-Arish A., Pandzic E., Goepp J., Matthes E., Hanrahan J.W., Wiseman P.W. (2015). Cholesterol modulates CFTR confinement in the plasma membrane of primary epithelial cells. Biophys. J..

[62] Abu-Arish A., Pandžić E., Kim D., Tseng H.W., Wiseman P.W., Hanrahan J.W. (2019). Agonists that stimulate secretion promote the recruitment of CFTR into membrane lipid microdomains. J. Gen. Physiol..

[63] Liessi N., Tomati V., Capurro V., Loberto N., Garcia-Aloy M., Franceschi P., Aureli M., Pedemonte N., Armirotti A. (2023). The combination elexacaftor/tezacaftor/ivacaftor (ETI) modulates the *de novo* synthethic pathway of ceramides in a genotype-independent manner. J. Cyst. Fibros..

[64] Schenkel M., Ravamehr-Lake D., Czerniak T., Saenz J.P., Krainer G., Schlierf M., Deber C.M. (2023). Impact of cholesterol and Lumacaftor on the folding of CFTR helical hairpins. Biochim. Biophys. Acta Biomembr..

[65] Centorame A., Dumut D.C., Youssef M., Ondra M., Kianicka I., Shah J., Paun R.A., Ozdian T., Hanrahan J.W., Gusev E. (2022). Treatment With LAU-7b Complements CFTR Modulator Therapy by Improving Lung Physiology and Normalizing Lipid Imbalance Associated With CF Lung Disease. Front. Pharmacol..

[66] Bae H., Kim B.R., Jung S., Le J., van der Heide D., Yu W., Park S.H., Hilkin B.M., Gansemer N.D., Powers L.S. (2024). Arteriovenous metabolomics in pigs reveals CFTR regulation of metabolism in multiple organs. J. Clin. Invest..

[67] Lonabaugh K., Li G., List R., Huang R., James A., Barros A., Somerville L., Albon D. (2023). Real world study on elexacaftor-tezacaftor-ivacaftor impact on cholesterol levels in adults with cystic fibrosis. Pharmacotherapy.

[68] Patel T., McBennett K., Sankararaman S., Schindler T., Sundaram K., Minich N.M., Malay S., Kutney K. (2024). Impact of elexacaftor/tezacaftor/ivacaftor on lipid and fat-soluble vitamin levels and association with body mass index. Pediatr. Pulmonol..

[69] Tindall A., Bass R., Maqbool A., Stallings V.A. (2023). Changes in nutrition and growth status in young children in the first 12 weeks of ivacaftor therapy. J. Cyst. Fibros..

[70] Tindall A., Bass R., Maqbool A., Stallings V.A. (2024). Impact of lumacaftor/ivacaftor on nutrition and growth in modulator-naïve children over 24 weeks. J. Cyst. Fibros..

[71] McDonald C., Yuzyuk T., Williams N., Meihls S., Asfour F. (2022). Essential fatty acid and lipoprotien status in pediatric cystic fibrosis: Impact of cystic fibrosis transmembrane conductoance regulator modulators. J. Cyst. Fibros..

[72] Slimmen L.J., Broos J.Y., Manai B.H., Estevao S.C., Giera M., Kooij G., Unger W.W.J., Janssens H.M. (2024). The Omega-6 lipid pathway shift is associated with neutrophil influx and structural lung damage in early cystic fibrosis lung disease. Clin. Transl. Immunol..

[73] Yuzyuk T., McDonald C.M., Zuromski L.M., De Biase I., Johnson L., Williams N., Meihls S., Asfour F. (2023). Improvement of lipid and lipoprotein profiles in children and adolescents with cystic fibrosis on CFTR modulator therapy. J. Cyst. Fibros..

[74] Keenan T.W., Morre D.J. (1970). Phopholipid class and fatty acid composition of Golgi apparatus isolated from rat liver and comparison with other cell fractions. Biochemistry.

[75] Hagve T.A. (1988). Effects of unsaturated fatty acids on cell membrane functions. Scand. J. Clin. Lab. Invest..

[76] Lee A.G. (2004). How lipids affect the activities of integral membrane proteins. Biochim. Biophys. Acta.

[77] Lee A.G. (2011). Lipid-protein interactions. Biochem. Soc. Trans..

[78] Kuo P.T., Huang N.N., Bassett D.R. (1962). The fatty acid composition of the serum chylomicrons and adipose tissue of children with cystic fibrosis of the pancreas. J. Pediatr..

[79] Underwood B.A., Denning C.R., Navab M. (1972). Polyunsaturated fatty acids and tocopherol levels in patients with cystic fibrosis. Ann. N. Y. Acad. Sci..

[80] Mischler E.H., Parrell S.W., Farrell P.M., Raynor W.J., Lemen R.J. (1986). Correction of linoleic acid deficiency in cystic fibrosis. Pediatr. Res..

[81] Freedman S.D., Blanco P.G., Zaman M.M., Shea J.C., Ollero M., Hopper I.K., Weed D.A., Gelrud A., Regan M.M., Laposata M. (2004). Association of cystic fibrosis with abnormalities in fatty acid metabolism. N. Engl. J. Med..

[82] Dowhan W. (1997). Molecular basis for membrane phospholipid diversity: why are there so many lipids?. Annu. Rev. Biochem..

[83] Harayama T., Riezman H. (2018). Understanding the diversity of membrane lipid composition. Nat. Rev. Mol. Cell Biol..

[84] Holman R.T. (1986). Nutritional and biochemical evidences of acyl interaction with respect to essential polyunsaturated fatty acids. Prog. Lipid Res..

[85] Korotkova M., Strandvik B. (2000). Essential fatty acid deficiency affects the fatty acid composition of the rat small intestinal and colonic mucosa differently. Biochim. Biophys. Acta.

[86] Steel D.M., Ryd W., Ascher H., Strandvik B. (2006). Abnormal fatty acid pattern in intestinal mucosa of children with celiac disease is not reflected in serum phospholipids. J. Pediatr. Gastroenterol. Nutr..

[87] Serhan C.N., Gupta S.K., Perretti M., Godson C., Brennan E., Li Y., Soehnlein O., Shimizu T., Werz O., Chiurchiù V. (2020). The Atlas of Inflammation Resolution (AIR). Mol. Aspect. Med..

[88] Talebi S., Day A.S., Khadem Rezaiyan M., Ranjbar G., Zarei M., Safarian M., Kianifar H.R. (2022). Fecal Calprotectin and Phenotype Severity in Patients with Cystic Fibrosis: A Systematic Review and Meta-Analysis. Pediatr. Gastroenterol. Hepatol. Nutr..

[89] Becker K.A., Riethmüller J., Zhang Y., Gulbins E. (2010). The role of sphingolipids and ceramide in pulmonary inflammation in cystic fibrosis. Open Respir. Med. J..

[90] Wojewodka G., De Sanctis J.B., Radzioch D. (2011). Ceramide in cystic fibrosis: a potential new target for therapeutic intervention. J. Lipids.

[91] Guilbault C., Wojewodka G., Saeed Z., Hajduch M., Matouk E., De Sanctis J.B., Radzioch D. (2009). Cystic fibrosis fatty acid imbalance is linked to ceramide deficiency and corrected by fenretinide. Am. J. Respir. Cell Mol. Biol..

[92] Bonfield T.L. (2020). Membrane Lipids and CFTR: The Yin/Yang of Efficient Ceramide Metabolism. Am. J. Respir. Crit. Care Med..

[93] Chanson M. (2009). [Cystic fibrosis, fatty acid imbalance, ceramide and lipid rafts]. Arch. Pediatr..

[94] Brodlie M., Lordan J.L., Ward C. (2011). Ceramide and cystic fibrosis lung disease. Am. J. Respir. Crit. Care Med..

[95] Kleuser B., Schumacher F., Gulbins E. (2023). New Therapeutic Options in Pulmonal Diseases: Sphingolipids and Modulation of Sphingolipid Metabolism. Handb. Exp. Pharmacol..

[96] Hamai H., Keyserman F., Quittell L.M., Worgall T.S. (2009). Defective CFTR increases synthesis and mass of sphingolipids that modulate membrane composition and lipid signaling. J. Lipid Res..

[97] Garić D., Dumut D.C., Shah J., De Sanctis J.B., Radzioch D. (2020). The role of essential fatty acids in cystic fibrosis and normalizing effect of fenretinide. Cell. Mol. Life Sci..

[98] Bal Topcu D., Er B., Ozcan F., Aslan M., Coplu L., Lay I., Oztas Y. (2023). Decreased plasma levels of sphingolipids and total cholesterol in adult cystic fibrosis patients. Prostaglandins Leukot. Essent. Fatty Acids.

[99] Amato F., Castaldo A., Castaldo G., Cernera G., Corso G., Ferrari E., Gelzo M., Monzani R., Villella V.R., Raia V. (2021). Impaired cholesterol metabolism in the mouse model of cystic fibrosis. A preliminary study. PLoS One.

[100] Cianciola N.L., Carlin C.R., Kelley T.J. (2011). Molecular pathways for intracellular cholesterol accumulation: common pathogenic mechanisms in Niemann-Pick disease Type C and cystic fibrosis. Arch. Biochem. Biophys..

[101] Cui G., Cottrill K.A., Strickland K.M., Mashburn S.A., Koval M., McCarty N.A. (2021). Alteration of Membrane Cholesterol Content Plays a Key Role in Regulation of Cystic Fibrosis Transmembrane Conductance Regulator Channel Activity. Front. Physiol..

[102] Chin S., Ramjeesingh M., Hung M., Ereño-Oreba J., Cui H., Laselva O., Julien J.P., Bear C.E. (2019). Cholesterol Interaction Directly Enhances Intrinsic Activity of the Cystic Fibrosis Transmembrane Conductance Regulator (CFTR). Cells.

[103] White N.M., Jiang D., Burgess J.D., Bederman I.R., Previs S.F., Kelley T.J. (2007). Altered cholesterol homeostasis in cultured and *in vivo* models of cystic fibrosis. Am. J. Physiol. Lung Cell Mol. Physiol..

[104] Fang D., West R.H., Manson M.E., Ruddy J., Jiang D., Previs S.F., Sonawane N.D., Burgess J.D., Kelley T.J. (2010). Increased plasma membrane cholesterol in cystic fibrosis cells correlates with CFTR genotype and depends on *de novo* cholesterol synthesis. Respir. Res..

[105] Gelzo M., Sica C., Elce A., Dello Russo A., Iacotucci P., Carnovale V., Raia V., Salvatore D., Corso G., Castaldo G. (2016). Reduced absorption and enhanced synthesis of cholesterol in patients with cystic fibrosis: a preliminary study of plasma sterols. Clin. Chem. Lab. Med..

[106] Lu B., Li L., Schneider M., Hodges C.A., Cotton C.U., Burgess J.D., Kelley T.J. (2019). Electrochemical measurement of membrane cholesterol correlates with CFTR function and is HDAC6-dependent. J. Cyst. Fibros..

[107] Rong X., Albert C.J., Hong C., Duerr M.A., Chamberlain B.T., Tarling E.J., Ito A., Gao J., Wang B., Edwards P.A. (2013). LXRs regulate ER stress and inflammation through dynamic modulation of membrane phospholipid composition. Cell Metabol..

[108] Strandvik B. (2010). Fatty acid metabolism in cystic fibrosis. Prostaglandins Leukot. Essent. Fatty Acids.

[109] Biggemann B., Laryea M.D., Schuster A., Griese M., Reinhardt D., Bremer H.J. (1988). Status of plasma and erythrocyte fatty acids and vitamin A and E in young children with cystic fibrosis. Scand. J. Gastroenterol. Suppl..

[110] Christophe A.B., Warwick W.J., Holman R.T. (1994). Serum fatty acid profiles in cystic fibrosis patients and their parents. Lipids.

[111] Lloyd-Still J.D., Johnson S.B., Holman R.T. (1991). Essential fatty acid status and fluidity of plasma phospholipids in cystic fibrosis infants. Am. J. Clin. Nutr..

[112] Rogiers V., Crokaert R., Vis H.L. (1980). Altered phospholipid composition and changed fatty acid pattern of the various phospholipid fractions of red cell membranes of cystic fibrosis children with pancreatic insufficiency. Clin. Chim. Acta.

[113] Rogiers V., Dab I., Crokaert R., Vis H.L. (1980). Long chain non-esterified fatty acid pattern in plasma of cystic fibrosis patients and their parents. Pediatr. Res..

[114] Rogiers V., Vercruysse A., Dab I., Baran D. (1983). Abnormal fatty acid pattern of the plasma cholesterol ester fraction in cystic fibrosis patients with and without pancreatic insufficiency. Eur. J. Pediatr..

[115] Rogiers V., Vercruysse A., Dab I., Crokaert R., Vis H.L. (1984). Fatty acid pattern of platelet phospholipids in cystic fibrosis. Eur. J. Pediatr..

[116] Maqbool A., Schall J.I., Garcia-Espana J.F., Zemel B.S., Strandvik B., Stallings V.A. (2008). Serum linoleic acid status as a clinical indicator of essential fatty acid status in children with cystic fibrosis. J. Pediatr. Gastroenterol. Nutr..

[117] Coste T.C., Deumer G., Reychler G., Lebecque P., Wallemacq P., Leal T. (2008). Influence of pancreatic status and sex on polyunsaturated fatty acid profiles in cystic fibrosis. Clin. Chem..

[118] Colombo C., Bennato V., Costantini D., Valmarana L., Daccò V., Zazzeron L., Ghisleni D., Bruzzese M.G., Scaglioni S., Riva E., Agostoni C. (2006). Dietary and circulating polyunsaturated fatty acids in cystic fibrosis: are they related to clinical outcomes?. J. Pediatr. Gastroenterol. Nutr..

[119] Walkowiak J., Lisowska A., Blaszczynski M., Przyslawski J., Walczak M. (2007). Polyunsaturated fatty acids in cystic fibrosis are related to nutrition and clinical expression of the disease. J. Pediatr. Gastroenterol. Nutr..

[120] Lai H.C., Kosorok M.R., Laxova A., Davis L.A., FitzSimmon S.C., Farrell P.M. (2000). Nutritional status of patients with cystic fibrosis with meconium ileus: a comparison with patients without meconium ileus and diagnosed early through neonatal screening. Pediatrics.

[121] Olveira G., Dorado A., Olveira C., Padilla A., Rojo-Martínez G., García-Escobar E., Gaspar I., Gonzalo M., Soriguer F. (2006). Serum phospholipid fatty acid profile and dietary intake in an adult Mediterranean population with cystic fibrosis. Br. J. Nutr..

[122] Coste T.C., Armand M., Lebacq J., Lebecque P., Wallemacq P., Leal T. (2007). An overview of monitoring and supplementation of omega 3 fatty acids in cystic fibrosis. Clin. Biochem..

[123] Rosenlund M.L., Selekman J.A., Kim H.K., Kritchevsky D. (1977). Dietary essential fatty acids in cystic fibrosis. Pediatrics.

[124] Rivers J.P., Hassam A.G. (1975). Defective essential-fatty-acid metabolism in cystic fibrosis. Lancet.

[125] Farrell P.M., Mischler E.H., Engle M.J., Brown D.J., Lau S.M. (1985). Fatty acid abnormalities in cystic fibrosis. Pediatr. Res..

[126] Uc A., Strandvik B., Yao J., Liu X., Yi Y., Sun X., Welti R., Engelhardt J.F., Norris A.W. (2022). The fatty acid imbalance of cystic fibrosis exists at birth independent of feeding in pig and ferret models. Clin. Sci. (Lond.).

[127] Maqbool A., Schall J.I., Gallagher P.R., Zemel B.S., Strandvik B., Stallings V.A. (2012). Relation between dietary fat intake type and serum fatty acid status in children with cystic fibrosis. J. Pediatr. Gastroenterol. Nutr..

[128] Van Biervliet S., Vanbillemont G., Van Biervliet J.P., Declercq D., Robberecht E., Christophe A. (2007). Relation between fatty acid composition and clinical status or genotype in cystic fibrosis patients. Ann. Nutr. Metab..

[129] Shrestha N., McCarron A., Rout-Pitt N., Donnelley M., Parsons D.W., Hryciw D.H. (2022). Essential Fatty Acid Deficiency in Cystic Fibrosis Disease Progression: Role of Genotype and Sex. Nutrients.

[130] Van Biervliet S., Van Biervliet J.P., Robberecht E., Christophe A. (2010). Fatty acid composition of serum phospholipids in cystic fibrosis (CF) patients with or without CF related liver disease. Clin. Chem. Lab. Med..

[131] Drzymała-Czyż S., Szczepanik M., Krzyżanowska P., Duś-Żuchowska M., Pogorzelski A., Sapiejka E., Juszczak P., Lisowska A., Koletzko B., Walkowiak J. (2017). Serum Phospholipid Fatty Acid Composition in Cystic Fibrosis Patients with and without Liver Cirrhosis. Ann. Nutr. Metab..

[132] Risé P., Volpi S., Colombo C., Padoan R.F., D'Orazio C., Ghezzi S., Melotti P., Bennato V., Agostoni C., Assael B.M., Galli C. (2010). Whole blood fatty acid analysis with micromethod in cystic fibrosis and pulmonary disease. J. Cyst. Fibros..

[133] Holman R.T. (1960). The ratio of trienoic: tetraenoic acids in tissue lipids as a measure of essential fatty acid requirement. J. Nutr..

[134] Batal I., Ericsoussi M.B., Cluette-Brown J.E., O'Sullivan B.P., Freedman S.D., Savaille J.E., Laposata M. (2007). Potential utility of plasma fatty acid analysis in the diagnosis of cystic fibrosis. Clin. Chem..

[135] Strandvik B. (1989). Relation between essential fatty acid metabolism and gastrointestinal symptoms in cystic fibrosis. Acta Paediatr. Scand. Suppl..

[136] Strandvik B., Bracco U., Deckelbaum R. (1992). Polyunsaturated Fatty Acids in Human Nutrition.

[137] Wheelock C.E., Strandvik B. (2020). Abnormal n-6 fatty acid metabolism in cystic fibrosis contributes to pulmonary symptoms. Prostaglandins Leukot. Essent. Fatty Acids.

[138] Rett B.S., Whelan J. (2011). Increasing dietary linoleic acid does not increase tissue arachidonic acid content in adults consuming Western-type diets: a systematic review. Nutr. Metab..

[139] Johnson G.H., Fritsche K. (2012). Effect of dietary linoleic acid on markers of inflammation in healthy persons: a systematic review of randomized controlled trials. J. Acad. Nutr. Diet..

[140] Liou Y.A., King D.J., Zibrik D., Innis S.M. (2007). Decreasing linoleic acid with constant alpha-linolenic acid in dietary fats increases (n-3) eicosapentaenoic acid in plasma phospholipids in healthy men. J. Nutr..

[141] Adam O., Wolfram G., Zöllner N. (2003). Influence of dietary linoleic acid intake with different fat intakes on arachidonic acid concentrations in plasma and platelet lipids and eicosanoid biosynthesis in female volunteers. Ann. Nutr. Metab..

[142] Babirekere-Iriso E., Lauritzen L., Mortensen C.G., Rytter M.J.H., Mupere E., Namusoke H., Michaelsen K.F., Briend A., Stark K.D., Metherel A.H., Friis H. (2016). Essential fatty acid composition and correlates in children with severe acute malnutrition. Clin. Nutr. ESPEN.

[143] Walkowiak J., Wilczynski M., Boleslawska I., Krawczynski M., Korzon M., Przyslawski J. (2003). The predominance of omega-6 polyunsaturated fatty acids in cystic fibrosis despite low arachidonic acid levels. Acta Paediatr..

[144] Shoff S.M., Ahn H.Y., Davis L., Lai H. (2006). Temporal associations among energy intake, plasma linoleic acid, and growth improvement in response to treatment initiation after diagnosis of cystic fibrosis. Pediatrics.

[145] Freedman S.D., Katz M.H., Parker E.M., Laposata M., Urman M.Y., Alvarez J.G. (1999). A membrane lipid imbalance plays a role in the phenotypic expression of cystic fibrosis in cftr(-/-) mice. Proc. Natl. Acad. Sci. USA.

[146] Beharry S., Ackerley C., Corey M., Kent G., Heng Y.M., Christensen H., Luk C., Yantiss R.K., Nasser I.A., Zaman M. (2007). Long-term docosahexaenoic acid therapy in a congenic murine model of cystic fibrosis. Am. J. Physiol. Gastrointest. Liver Physiol..

[147] Watson H., Stackhouse C. (2020). Omega-3 fatty acid supplementation for cystic fibrosis. Cochrane Database Syst. Rev..

[148] Sohouli M.H., Magalhães E., Ghahramani S., Nasresfahani M., Ezoddin N., Sharifi P., Rohani P. (2023). Impact of omega-3 supplementation on children and adolescents patients with cystic fibrosis: A systematic review and meta-analysis of randomized-controlled trials. Pediatr. Pulmonol..

[149] De Vizia B., Raia V., Spano C., Pavlidis C., Coruzzo A., Alessio M. (2003). Effect of an 8-month treatment with omega-3 fatty acids (eicosapentaenoic and docosahexaenoic) in patients with cystic fibrosis. JPEN J. Parenter. Enteral Nutr..

[150] Strandvik B., WK O.N., Ali M.A., Hammar U. (2018). Low linoleic and high docosahexaenoic acids in a severe phenotype of transgenic cystic fibrosis mice. Exp. Biol. Med. (Maywood, NJ, U. S.).

[151] Innis S.M., Davidson A.G., Chen A., Dyer R., Melnyk S., James S.J. (2003). Increased plasma homocysteine and S-adenosylhomocysteine and decreased methionine is associated with altered phosphatidylcholine and phosphatidylethanolamine in cystic fibrosis. J. Pediatr..

[152] Philippe R., Urbach V. (2018). Specialized Pro-Resolving Lipid Mediators in Cystic Fibrosis. Int. J. Mol. Sci..

[153] Recchiuti A., Patruno S., Plebani R., Romano M. (2020). The Resolution Approach to Cystic Fibrosis Inflammation. Front. Pharmacol..

[154] Grassmé H., Riethmüller J., Gulbins E. (2013). Ceramide in cystic fibrosis. Handb. Exp. Pharmacol..

[155] Garić D., De Sanctis J.B., Shah J., Dumut D.C., Radzioch D. (2019). Biochemistry of very-long-chain and long-chain ceramides in cystic fibrosis and other diseases: The importance of side chain. Prog. Lipid Res..

[156] Teichgräber V., Ulrich M., Endlich N., Riethmüller J., Wilker B., De Oliveira-Munding C.C., van Heeckeren A.M., Barr M.L., von Kürthy G., Schmid K.W. (2008). Ceramide accumulation mediates inflammation, cell death and infection susceptibility in cystic fibrosis. Nat. Med..

[157] Dobi D., Loberto N., Bassi R., Pistocchi A., Lunghi G., Tamanini A., Aureli M. (2023). Cross-talk between CFTR and sphingolipids in cystic fibrosis. FEBS Open Bio.

[158] Garić D., De Sanctis J.B., Wojewodka G., Houle D., Cupri S., Abu-Arish A., Hanrahan J.W., Hajduch M., Matouk E., Radzioch D. (2017). Fenretinide differentially modulates the levels of long- and very long-chain ceramides by downregulating Cers5 enzyme: evidence from bench to bedside. J. Mol. Med. (Berl.).

[159] Youssef M., De Sanctis J.B., Shah J., Dumut D.C., Hajduch M., Petrof B.J., Radzioch D. (2020). Age-Dependent Progression in Lung Pathophysiology can be Prevented by Restoring Fatty Acid and Ceramide Imbalance in Cystic Fibrosis. Lung.

[160] White M.M., Geraghty P., Hayes E., Cox S., Leitch W., Alfawaz B., Lavelle G.M., McElvaney O.J., Flannery R., Keenan J. (2017). Neutrophil Membrane Cholesterol Content is a Key Factor in Cystic Fibrosis Lung Disease. EBioMedicine.

[161] Drzymała-Czyż S., Krzyżanowska-Jankowska P., Dziedzic K., Lisowska A., Kurek S., Goździk-Spychalska J., Kononets V., Woźniak D., Mądry E., Walkowiak J. (2021). Severe Genotype, Pancreatic Insufficiency and Low Dose of Pancreatic Enzymes Associate with Abnormal Serum Sterol Profile in Cystic Fibrosis. Biomolecules.

[162] Iuliano L., Monticolo R., Straface G., Zullo S., Galli F., Boaz M., Quattrucci S. (2009). Association of cholesterol oxidation and abnormalities in fatty acid metabolism in cystic fibrosis. Am. J. Clin. Nutr..

[163] Gabbi C., Warner M., Gustafsson J. (2014). Action mechanisms of Liver X Receptors. Biochem. Biophys. Res. Commun..

[164] Gabbi C., Kim H.J., Hultenby K., Bouton D., Toresson G., Warner M., Gustafsson J.A. (2008). Pancreatic exocrine insufficiency in LXRbeta-/- mice is associated with a reduction in aquaporin-1 expression. Proc. Natl. Acad. Sci. USA.

[165] Russo-Savage L., Schulman I.G. (2021). Liver X receptors and liver physiology. Biochim. Biophys. Acta, Mol. Basis Dis..

[166] Ulven S.M., Dalen K.T., Gustafsson J.A., Nebb H.I. (2005). LXR is crucial in lipid metabolism. Prostaglandins Leukot. Essent. Fatty Acids.

[167] Savant A.P., McColley S.A. (2023). Cystic fibrosis year in review 2019: Section 3 multisystem-based care and research. Pediatr. Pulmonol..

[168] De Boeck K. (2020). Cystic fibrosis in the year 2020: A disease with a new face. Acta Paediatr..

[169] Rowe S.M., Accurso F., Clancy J.P. (2007). Detection of cystic fibrosis transmembrane conductance regulator activity in early-phase clinical trials. Proc. Am. Thorac. Soc..

[170] Goubau C., Wilschanski M., Skalická V., Lebecque P., Southern K.W., Sermet I., Munck A., Derichs N., Middleton P.G., Hjelte L. (2009). Phenotypic characterisation of patients with intermediate sweat chloride values: towards validation of the European diagnostic algorithm for cystic fibrosis. Thorax.

[171] Heijerman H.G.M., McKone E.F., Downey D.G., Van Braeckel E., Rowe S.M., Tullis E., Mall M.A., Welter J.J., Ramsey B.W., McKee C.M. (2019). Efficacy and safety of the elexacaftor plus tezacaftor plus ivacaftor combination regimen in people with cystic fibrosis homozygous for the F508del mutation: a double-blind, randomised, phase 3 trial. Lancet.

[172] Davies J.C., Sermet-Gaudelus I., Naehrlich L., Harris R.S., Campbell D., Ahluwalia N., Short C., Haseltine E., Panorchan P., Saunders C. (2021). A phase 3, double-blind, parallel-group study to evaluate the efficacy and safety of tezacaftor in combination with ivacaftor in participants 6 through 11 years of age with cystic fibrosis homozygous for F508del or heterozygous for the F508del-CFTR mutation and a residual function mutation. J. Cyst. Fibros..

[173] Uluer A.Z., MacGregor G., Azevedo P., Indihar V., Keating C., Mall M.A., McKone E.F., Ramsey B.W., Rowe S.M., Rubenstein R.C. (2023). Safety and efficacy of vanzacaftor-tezacaftor-deutivacaftor in adults with cystic fibrosis: randomised, double-blind, controlled, phase 2 trials. Lancet Respir. Med..

[174] Kusoffsky E., Strandvik B., Troell S. (1983). Prospective study of fatty acid supplementation over 3 years in patients with cystic fibrosis. J. Pediatr. Gastroenterol. Nutr..

[175] Elliott R.B., Robinson P.G. (1975). Unusual clinical course in a child with cystic fibrosis treated with fat emulsion. Arch. Dis. Child..

[176] Elliott R.B. (1976). A therapeutic trial of fatty acid supplementation in cystic fibrosis. Pediatrics.

[177] Dodge J.A., Custance J.M., Goodchild M.C., Laing S.C., Vaughan M. (1990). Paradoxical effects of essential fatty acid supplementation on lipid profiles and sweat electrolytes in cystic fibrosis. Br. J. Nutr..

[178] Reddy M.M., Quinton P.M. (2003). Functional interaction of CFTR and ENaC in sweat glands. Pflügers Archiv.

[179] Boscardin E., Alijevic O., Hummler E., Frateschi S., Kellenberger S. (2016). The function and regulation of acid-sensing ion channels (ASICs) and the epithelial Na(+) channel (ENaC): IUPHAR Review 19. Br. J. Pharmacol..

[180] van Egmond A.W., Kosorok M.R., Koscik R., Laxova A., Farrell P.M. (1996). Effect of linoleic acid intake on growth of infants with cystic fibrosis. Am. J. Clin. Nutr..

[181] Lai H.C., Corey M., FitzSimmons S., Kosorok M.R., Farrell P.M. (1999). Comparison of growth status of patients with cystic fibrosis between the United States and Canada. Am. J. Clin. Nutr..

[182] Munck A., Boulkedid R., Weiss L., Foucaud P., Wizla-Derambure N., Reix P., Bremont F., Derelle J., Schroedt J., Alberti C. (2018). Nutritional Status in the First 2 Years of Life in Cystic Fibrosis Diagnosed by Newborn Screening. J. Pediatr. Gastroenterol. Nutr..

[183] Woestenenk J.W., Castelijns S.J., van der Ent C.K., Houwen R.H. (2014). Dietary intake in children and adolescents with cystic fibrosis. Clin. Nutr..

[184] Kastner-Cole D., Palmer C.N., Ogston S.A., Mehta A., Mukhopadhyay S. (2005). Overweight and obesity in deltaF508 homozygous cystic fibrosis. J. Pediatr..

[185] Kutney K.A., Sandouk Z., Desimone M., Moheet A. (2021). Obesity in cystic fibrosis. J. Clin. Transl. Endocrinol..

[186] Kim J., Lyman B., Savant A.P. (2023). Cystic fibrosis year in review 2022. Pediatr. Pulmonol..

[187] Rettammel A.L., Marcus M.S., Farrell P.M., Sondel S.A., Koscik R.E., Mischler E.H. (1995). Oral supplementation with a high-fat, high-energy product improves nutritional status and alters serum lipids in patients with cystic fibrosis. J. Am. Diet Assoc..

[188] Kindstedt-Arfwidson K., Strandvik B. (1988). Food intake in patients with cystic fibrosis on an ordinary diet. Scand. J. Gastroenterol. Suppl..

[189] Mohrhauer H., Holman T. (1967). Metabolism of linoleic acid in relation to dietary saturated fatty acids in the rat. J. Nutr..

[190] Cunnane S.C., Anderson M.J. (1997). Pure linoleate deficiency in the rat: influence on growth, accumulation of n-6 polyunsaturates, and [1-14C]linoleate oxidation. J. Lipid Res..

[191] Dodge J.A. (1985). The nutritional state and nutrition. Acta Paediatr. Scand. Suppl..

[192] Darrah R., Nelson R., Damato E.G., Decker M., Matthews A., Hodges C.A. (2016). Growth Deficiency in Cystic Fibrosis Is Observable at Birth and Predictive of Early Pulmonary Function. Biol. Res. Nurs..

[193] Sanders D.B., Zhang Z., Farrell P.M., Lai H.J. (2018). Early life growth patterns persist for 12 years and impact pulmonary outcomes in cystic fibrosis. J. Cyst. Fibros..

[194] Zolin A., Adamoli A., Bakkeheim E., van Rens J., Prasad V., Fox A., Krasnyk M., Mayor S.L., Naehrlich L., Gkolia P. (2024). ECFS Patient Registry Annual Report 2022. http://www.ecfs.eu.

[195] Faro A., Marshall B., Brown W., Elbert A., Beres C., Cromwell E., Ostrenga J., Petren K., Rizvi S., Wu R., Yuth M. (2024). Cystic Fibrosis Foundation Patient Registry Annual Data Report 2023. http://www.cff.org.

[196] Lai H.J., Shoff S.M., Farrell P.M. (2009). Recovery of birth weight z score within 2 years of diagnosis is positively associated with pulmonary status at 6 years of age in children with cystic fibrosis. Pediatrics.

[197] Li Z., Lai H.J., Kosorok M.R., Laxova A., Rock M.J., Splaingard M.L., Farrell P.M. (2004). Longitudinal pulmonary status of cystic fibrosis children with meconium ileus. Pediatr. Pulmonol..

[198] Steinkamp G., Demmelmair H., Rühl-Bagheri I., von der Hardt H., Koletzko B. (2000). Energy supplements rich in linoleic acid improve body weight and essential fatty acid status of cystic fibrosis patients. J. Pediatr. Gastroenterol. Nutr..

[199] Switzer M., Rice J., Rice M., Hardin D.S. (2009). Insulin-like growth factor-I levels predict weight, height and protein catabolism in children and adolescents with cystic fibrosis. J. Pediatr. Endocrinol. Metab..

[200] Bessich J.L., Nymon A.B., Moulton L.A., Dorman D., Ashare A. (2013). Low levels of insulin-like growth factor-1 contribute to alveolar macrophage dysfunction in cystic fibrosis. J. Immunol..

[201] Arumugam R., LeBlanc A., Seilheimer D.K., Hardin D.S. (1998). Serum leptin and IGF-I levels in cystic fibrosis. Endocr. Res..

[202] Laursen E.M., Juul A., Lanng S., Høiby N., Koch C., Müller J., Skakkebaek N.E. (1995). Diminished concentrations of insulin-like growth factor I in cystic fibrosis. Arch. Dis. Child..

[203] Rogan M.P., Reznikov L.R., Pezzulo A.A., Gansemer N.D., Samuel M., Prather R.S., Zabner J., Fredericks D.C., McCray P.B., Welsh M.J., Stoltz D.A. (2010). Pigs and humans with cystic fibrosis have reduced insulin-like growth factor 1 (IGF1) levels at birth. Proc. Natl. Acad. Sci. USA.

[204] Taylor A.M., Bush A., Thomson A., Oades P.J., Marchant J.L., Bruce-Morgan C., Holly J., Ahmed L., Dunger D.B. (1997). Relation between insulin-like growth factor-I, body mass index, and clinical status in cystic fibrosis. Arch. Dis. Child..

[205] Sermet-Gaudelus I., Souberbielle J.C., Azhar I., Ruiz J.C., Magnine P., Colomb V., Le Bihan C., Folio D., Lenoir G. (2003). Insulin-like growth factor I correlates with lean body mass in cystic fibrosis patients. Arch. Dis. Child..

[206] Rosenberg L.A., Schluchter M.D., Parlow A.F., Drumm M.L. (2006). Mouse as a model of growth retardation in cystic fibrosis. Pediatr. Res..

[207] Stalvey M.S., Havasi V., Tuggle K.L., Wang D., Birket S., Rowe S.M., Sorscher E.J. (2017). Reduced bone length, growth plate thickness, bone content, and IGF-I as a model for poor growth in the CFTR-deficient rat. PLoS One.

[208] Phung O.J., Coleman C.I., Baker E.L., Scholle J.M., Girotto J.E., Makanji S.S., Chen W.T., Talati R., Kluger J., White C.M. (2010). Recombinant human growth hormone in the treatment of patients with cystic fibrosis. Pediatrics.

[209] Reisi M., Sharif Ahmadian N., Hashemipour M., Mostofizadeh N., Keivanfar M., Hashemi E. (2022). Evaluation of Growth Hormone Deficiency in Children with Cystic Fibrosis. Adv. Biomed. Res..

[210] Thaker V., Carter B., Putman M. (2021). Recombinant growth hormone therapy for cystic fibrosis in children and young adults. Cochrane Database Syst. Rev..

[211] Kjellberg E., Roswall J., Bergman S., Strandvik B., Dahlgren J. (2018). Serum n-6 and n-9 Fatty Acids Correlate With Serum IGF-1 and Growth Up to 4 Months of Age in Healthy Infants. J. Pediatr. Gastroenterol. Nutr..

[212] Korotkova M., Ohlsson C., Gabrielsson B., Hanson L.A., Strandvik B. (2005). Perinatal essential fatty acid deficiency influences body weight and bone parameters in adult male rats. Biochim. Biophys. Acta.

[213] Korotkova M., Ohlsson C., Hanson L.A., Strandvik B. (2004). Dietary n-6:n-3 fatty acid ratio in the perinatal period affects bone parameters in adult female rats. Br. J. Nutr..

[214] Fried M.D., Durie P.R., Tsui L.C., Corey M., Levison H., Pencharz P.B. (1991). The cystic fibrosis gene and resting energy expenditure. J. Pediatr..

[215] O'Rawe A., McIntosh I., Dodge J.A., Brock D.J., Redmond A.O., Ward R., Macpherson A.J. (1992). Increased energy expenditure in cystic fibrosis is associated with specific mutations. Clin. Sci. (Lond.).

[216] Zemel B.S., Kawchak D.A., Cnaan A., Zhao H., Scanlin T.F., Stallings V.A. (1996). Prospective evaluation of resting energy expenditure, nutritional status, pulmonary function, and genotype in children with cystic fibrosis. Pediatr. Res..

[217] Moudiou T., Galli-Tsinopoulou A., Vamvakoudis E., Nousia-Arvanitakis S. (2007). Resting energy expenditure in cystic fibrosis as an indicator of disease severity. J. Cyst. Fibros..

[218] Magoffin A., Allen J.R., McCauley J., Gruca M.A., Peat J., Van Asperen P., Gaskin K. (2008). Longitudinal analysis of resting energy expenditure in patients with cystic fibrosis. J. Pediatr..

[219] Barclay A., Allen J.R., Blyler E., Yap J., Gruca M.A., Asperen P.V., Cooper P., Gaskin K.J. (2007). Resting energy expenditure in females with cystic fibrosis: is it affected by puberty?. Eur. J. Clin. Nutr..

[220] Groleau V., Schall J.I., Dougherty K.A., Latham N.E., Maqbool A., Mascarenhas M.R., Stallings V.A. (2014). Effect of a dietary intervention on growth and energy expenditure in children with cystic fibrosis. J. Cyst. Fibros..

[221] Moudiou T., Galli-Tsinopoulou A., Nousia-Arvanitakis S. (2007). Effect of exocrine pancreatic function on resting energy expenditure in cystic fibrosis. Acta Paediatr..

[222] Rafael J., Patzelt J., Schäfer H., Elmadfa I. (1984). The effect of essential fatty acid deficiency on basal respiration and function of liver mitochondria in rats. J. Nutr..

[223] van der Veen J.N., Kennelly J.P., Wan S., Vance J.E., Vance D.E., Jacobs R.L. (2017). The critical role of phosphatidylcholine and phosphatidylethanolamine metabolism in health and disease. Biochim. Biophys. Acta Biomembr..

[224] Mårtensson C.U., Doan K.N., Becker T. (2017). Effects of lipids on mitochondrial functions. Biochim. Biophys. Acta Mol. Cell Biol. Lipids.

[225] Antigny F., Girardin N., Raveau D., Frieden M., Becq F., Vandebrouck C. (2009). Dysfunction of mitochondria Ca2+ uptake in cystic fibrosis airway epithelial cells. Mitochondrion.

[226] Favia M., de Bari L., Bobba A., Atlante A. (2019). An Intriguing Involvement of Mitochondria in Cystic Fibrosis. J. Clin. Med..

[227] Joseloff E., Sha W., Bell S.C., Wetmore D.R., Lawton K.A., Milburn M.V., Ryals J.A., Guo L., Muhlebach M.S. (2014). Serum metabolomics indicate altered cellular energy metabolism in children with cystic fibrosis. Pediatr. Pulmonol..

[228] Atlante A., Favia M., Bobba A., Guerra L., Casavola V., Reshkin S.J. (2016). Characterization of mitochondrial function in cells with impaired cystic fibrosis transmembrane conductance regulator (CFTR) function. J. Bioenerg. Biomembr..

[229] Kleme M.L., Sané A., Garofalo C., Seidman E., Brochiero E., Berthiaume Y., Levy E. (2018). CFTR Deletion Confers Mitochondrial Dysfunction and Disrupts Lipid Homeostasis in Intestinal Epithelial Cells. Nutrients.

[230] Aravamudan B., Thompson M.A., Pabelick C.M., Prakash Y.S. (2013). Mitochondria in lung diseases. Expet Rev. Respir. Med..

[231] Valdivieso A.G., Santa-Coloma T.A. (2013). CFTR activity and mitochondrial function. Redox Biol..

[232] Paradies G., Paradies V., Ruggiero F.M., Petrosillo G. (2019). Role of Cardiolipin in Mitochondrial Function and Dynamics in Health and Disease: Molecular and Pharmacological Aspects. Cells.

[233] Kunzelmann K., Schreiber R., Hadorn H.B. (2017). Bicarbonate in cystic fibrosis. J. Cyst. Fibros..

[234] Gibson-Corley K.N., Meyerholz D.K., Engelhardt J.F. (2016). Pancreatic pathophysiology in cystic fibrosis. J. Pathol..

[235] Uc A., Giriyappa R., Meyerholz D.K., Griffin M., Ostedgaard L.S., Tang X.X., Abu-El-Haija M., Stoltz D.A., Ludwig P., Pezzulo A. (2012). Pancreatic and biliary secretion are both altered in cystic fibrosis pigs. Am. J. Physiol. Gastrointest. Liver Physiol..

[236] Rotti P.G., Evans I.A., Zhang Y., Liang B., Cunicelli N., O'Malley Y., Norris A.W., Uc A., Engelhardt J.F. (2022). Lack of CFTR alters the ferret pancreatic ductal epithelial secretome and cellular proteome: Implications for exocrine/endocrine signaling. J. Cyst. Fibros..

[237] Hjelte L., Ahrén B., Andrén-Sandberg A., Böttcher G., Strandvik B. (1990). Pancreatic function in the essential fatty acid deficient rat. Metabolism.

[238] Hou W., Arita Y., Morisset J. (1997). Endogenous arachidonic acid release and pancreatic amylase secretion. Pancreas.

[239] Marshall P.J., Dixon J.F., Hokin L.E. (1982). Prostaglandin E2 derived from phosphatidylinositol breakdown in the exocrine pancreas facilitates secretion by an action on the ducts. J. Pharmacol. Exp. Therapeut..

[240] Wooten M.W., Wrenn R.W. (1988). Linoleic acid is a potent activator of protein kinase C type III-alpha isoform in pancreatic acinar cells; its role in amylase secretion. Biochem. Biophys. Res. Commun..

[241] Ortiz-Placín C., Castillejo-Rufo A., Estarás M., González A. (2023). Membrane Lipid Derivatives: Roles of Arachidonic Acid and Its Metabolites in Pancreatic Physiology and Pathophysiology. Molecules.

[242] Lai M.C., Teng T.H., Yang C. (2013). The natural PPAR agonist linoleic acid stimulated insulin release in the rat pancreas. J. Vet. Med. Sci..

[243] Hjelte L., Nilsson K., Moen I.E., Lindblad A., Mared L., Pressler T., Fluge G., Consortium f.t.S.C.S. (2008). Linoleic acid but not EPA and DHA correlates to prognostic markers in Scandinavian CF patients. J. Cyst. Fibros..

[244] Colomba J., Boudreau V., Lehoux-Dubois C., Desjardins K., Coriati A., Tremblay F., Rabasa-Lhoret R. (2019). The main mechanism associated with progression of glucose intolerance in older patients with cystic fibrosis is insulin resistance and not reduced insulin secretion capacity. J. Cyst. Fibros..

[245] Schmid K., Fink K., Holl R.W., Hebestreit H., Ballmann M. (2014). Predictors for future cystic fibrosis-related diabetes by oral glucose tolerance test. J. Cyst. Fibros..

[246] Belury M.A., Cole R.M., Snoke D.B., Banh T., Angelotti A. (2018). Linoleic acid, glycemic control and Type 2 diabetes. Prostaglandins Leukot. Essent. Fatty Acids.

[247] Olesen H.V., Drevinek P., Gulmans V.A., Hatziagorou E., Jung A., Mei-Zahav M., Stojnic N., Thomas M., Zolin A. (2020). Cystic fibrosis related diabetes in Europe: Prevalence, risk factors and outcome; Olesen et al. J. Cyst. Fibros..

[248] Fontés G., Ghislain J., Benterki I., Zarrouki B., Trudel D., Berthiaume Y., Poitout V. (2015). The ΔF508 Mutation in the Cystic Fibrosis Transmembrane Conductance Regulator Is Associated With Progressive Insulin Resistance and Decreased Functional β-Cell Mass in Mice. Diabetes.

[249] Uc A., Olivier A.K., Griffin M.A., Meyerholz D.K., Yao J., Abu-El-Haija M., Buchanan K.M., Vanegas Calderón O.G., Abu-El-Haija M., Pezzulo A.A. (2015). Glycaemic regulation and insulin secretion are abnormal in cystic fibrosis pigs despite sparing of islet cell mass. Clin. Sci. (Lond.).

[250] Persaud S.J., Muller D., Belin V.D., Kitsou-Mylona I., Asare-Anane H., Papadimitriou A., Burns C.J., Huang G.C., Amiel S.A., Jones P.M. (2007). The role of arachidonic acid and its metabolites in insulin secretion from human islets of langerhans. Diabetes.

[251] Das U.N. (2013). Arachidonic acid and lipoxin A4 as possible endogenous anti-diabetic molecules. Prostaglandins Leukot. Essent. Fatty Acids.

[252] Karp C.L., Flick L.M., Park K.W., Softic S., Greer T.M., Keledjian R., Yang R., Uddin J., Guggino W.B., Atabani S.F. (2004). Defective lipoxin-mediated anti-inflammatory activity in the cystic fibrosis airway. Nat. Immunol..

[253] Gao Y., Su J., Zhang Y., Chan A., Sin J.H., Wu D., Min K., Gronert K. (2018). Dietary DHA amplifies LXA(4) circuits in tissues and lymph node PMN and is protective in immune-driven dry eye disease. Mucosal Immunol..

[254] Hallberg K., Abrahamsson H., Dalenbäck J., Fändriks L., Strandvik B. (2001). Gastric secretion in cystic fibrosis in relation to the migrating motor complex. Scand. J. Gastroenterol..

[255] Kollberg B., Aly A., Johansson C. (1981). Protection of the rat gastric mucosa by prostaglandin E2: possible relation to stimulation of the alkaline secretion. Acta Physiol. Scand..

[256] Johansson C., Aly A., Befrits R., Smedfors B., Uribe A. (1985). Protection of the gastroduodenal mucosa by prostaglandins. Scand. J. Gastroenterol. Suppl..

[257] Strandvik B., Svensson E., Seyberth H.W. (1996). Prostanoid biosynthesis in patients with cystic fibrosis. Prostaglandins Leukot. Essent. Fatty Acids.

[258] Jabr S., Gartner S., Milne G.L., Roca-Ferrer J., Casas J., Moreno A., Gelpí E., Picado C. (2013). Quantification of major urinary metabolites of PGE2 and PGD2 in cystic fibrosis: correlation with disease severity. Prostaglandins Leukot. Essent. Fatty Acids.

[259] Littlewood J.M., Wolfe S.P. (2000). Control of malabsorption in cystic fibrosis. Paediatr. Drugs.

[260] van Elburg R.M., Uil J.J., van Aalderen W.M., Mulder C.J., Heymans H.S. (1996). Intestinal permeability in exocrine pancreatic insufficiency due to cystic fibrosis or chronic pancreatitis. Pediatr. Res..

[261] Hallberg K., Grzegorczyk A., Larson G., Strandvik B. (1997). Intestinal permeability in cystic fibrosis in relation to genotype. J. Pediatr. Gastroenterol. Nutr..

[262] Duranthon V., Frémont L., Léger C.L. (1991). Effect of essential fatty acid deficiency on lipid composition of basolateral plasma membrane of pig intestinal mucosal cells. Lipids.

[263] Christon R., Meslin J.C., Thévenoux J., Linard A., Léger C.L., Delpal S. (1991). Effects of a low dietary linoleic acid level on intestinal morphology and enterocyte brush border membrane lipid composition. Reprod. Nutr. Dev..

[264] Peretti N., Marcil V., Drouin E., Levy E. (2005). Mechanisms of lipid malabsorption in Cystic Fibrosis: the impact of essential fatty acids deficiency. Nutr. Metab..

[265] Hardcastle J., Harwood M.D., Taylor C.J. (2004). Absorption of taurocholic acid by the ileum of normal and transgenic DeltaF508 cystic fibrosis mice. J. Pharm. Pharmacol..

[266] Shrestha N., Rout-Pitt N., McCarron A., Jackson C.A., Bulmer A.C., McAinch A.J., Donnelley M., Parsons D.W., Hryciw D.H. (2023). Changes in Essential Fatty Acids and Ileal Genes Associated with Metabolizing Enzymes and Fatty Acid Transporters in Rodent Models of Cystic Fibrosis. Int. J. Mol. Sci..

[267] Steagall W.K., Elmer H.L., Brady K.G., Kelley T.J. (2000). Cystic fibrosis transmembrane conductance regulator-dependent regulation of epithelial inducible nitric oxide synthase expression. Am. J. Respir. Cell Mol. Biol..

[268] De Lisle R.C., Meldi L., Flynn M., Jansson K. (2008). Altered eicosanoid metabolism in the cystic fibrosis mouse small intestine. J. Pediatr. Gastroenterol. Nutr..

[269] Brulet M., Seyer A., Edelman A., Brunelle A., Fritsch J., Ollero M., Laprévote O. (2010). Lipid mapping of colonic mucosa by cluster TOF-SIMS imaging and multivariate analysis in cftr knockout mice. J. Lipid Res..

[270] Sbarbati A., Bertini M., Catassi C., Gagliardini R., Osculati F. (1998). Ultrastructural lesions in the small bowel of patients with cystic fibrosis. Pediatr. Res..

[271] Liu J., Walker N.M., Ootani A., Strubberg A.M., Clarke L.L. (2015). Defective goblet cell exocytosis contributes to murine cystic fibrosis-associated intestinal disease. J. Clin. Invest..

[272] Stastna N., Kunovsky L., Svoboda M., Pokojova E., Homola L., Mala M., Gracova Z., Jerabkova B., Skrickova J., Trna J. (2024). Improved Nutritional Outcomes and Gastrointestinal Symptoms in Adult Cystic Fibrosis Patients Treated with Elexacaftor/Tezacaftor/Ivacaftor. Dig. Dis..

[273] Kunzelmann K., Mall M. (2002). Electrolyte transport in the mammalian colon: mechanisms and implications for disease. Physiol. Rev..

[274] Smyth R.L., Croft N.M., O'Hea U., Marshall T.G., Ferguson A. (2000). Intestinal inflammation in cystic fibrosis. Arch. Dis. Child..

[275] Enaud R., Hooks K.B., Barre A., Barnetche T., Hubert C., Massot M., Bazin T., Clouzeau H., Bui S., Fayon M. (2019). Intestinal Inflammation in Children with Cystic Fibrosis Is Associated with Crohn's-Like Microbiota Disturbances. J. Clin. Med..

[276] Parisi G.F., Papale M., Rotolo N., Aloisio D., Tardino L., Scuderi M.G., Di Benedetto V., Nenna R., Midulla F., Leonardi S. (2017). Severe disease in Cystic Fibrosis and fecal calprotectin levels. Immunobiology.

[277] Dhaliwal J., Leach S., Katz T., Nahidi L., Pang T., Lee J.M., Strachan R., Day A.S., Jaffe A., Ooi C.Y. (2015). Intestinal inflammation and impact on growth in children with cystic fibrosis. J. Pediatr. Gastroenterol. Nutr..

[278] De Lisle R.C., Borowitz D. (2013). The cystic fibrosis intestine. Cold Spring Harbor Perspect. Med..

[279] De Lisle R.C. (2014). Disrupted tight junctions in the small intestine of cystic fibrosis mice. Cell Tissue Res..

[280] Duong J.T., Pope C.E., Hayden H.S., Miller C., Salipante S.J., Rowe S.M., Solomon G.M., Nichols D., Hoffman L.R., Narkewicz M.R., Green N. (2024). Alterations in the fecal microbiota in patients with advanced cystic fibrosis liver disease after 6 months of elexacaftor/tezacaftor/ivacaftor. J. Cyst. Fibros..

[281] Dif F., Wu Y.Z., Burgel P.R., Ollero M., Leduc D., Aarbiou J., Borot F., Garcia-Verdugo I., Martin C., Chignard M. (2010). Critical role of cytosolic phospholipase A2{alpha} in bronchial mucus hypersecretion in CFTR-deficient mice. Eur. Respir. J..

[282] Greger R. (2000). Role of CFTR in the colon. Annu. Rev. Physiol..

[283] de Lisle R.C., Sewell R., Meldi L. (2010). Enteric circular muscle dysfunction in the cystic fibrosis mouse small intestine. Neuro Gastroenterol. Motil..

[284] de Freitas M.B., Moreira E.A.M., Tomio C., Moreno Y.M.F., Daltoe F.P., Barbosa E., Ludwig Neto N., Buccigrossi V., Guarino A. (2018). Altered intestinal microbiota composition, antibiotic therapy and intestinal inflammation in children and adolescents with cystic fibrosis. PLoS One.

[285] Anand S., Mande S.S. (2018). Diet, Microbiota and Gut-Lung Connection. Front. Microbiol..

[286] Zhang D., Li S., Wang N., Tan H.Y., Zhang Z., Feng Y. (2020). The Cross-Talk Between Gut Microbiota and Lungs in Common Lung Diseases. Front. Microbiol..

[287] Kinnman N., Lindblad A., Housset C., Buentke E., Scheynius A., Strandvik B., Hultcrantz R. (2000). Expression of cystic fibrosis transmembrane conductance regulator in liver tissue from patients with cystic fibrosis. Hepatology.

[288] Cohn J.A., Strong T.V., Picciotto M.R., Nairn A.C., Collins F.S., Fitz J.G. (1993). Localization of the cystic fibrosis transmembrane conductance regulator in human bile duct epithelial cells. Gastroenterology.

[289] Lukasiak A., Zajac M. (2021). The Distribution and Role of the CFTR Protein in the Intracellular Compartments. Membranes.

[290] Craig J.M., Haddad H., Shwachman H. (1957). The pathological changes in the liver in cystic fibrosis of the pancreas. AMA J. Dis. Child..

[291] Lindblad A., Glaumann H., Strandvik B. (1999). Natural history of liver disease in cystic fibrosis. Hepatology.

[292] Jørgensen M.H., Ott P., Michaelsen K.F., Porsgaard T., Jensen F., Lanng S. (2012). Long-chain PUFA in granulocytes, mononuclear cells, and RBC in patients with cystic fibrosis: relation to liver disease. J. Pediatr. Gastroenterol. Nutr..

[293] Strandvik B., Hultcrantz R. (1994). Liver function and morphology during long-term fatty acid supplementation in cystic fibrosis. Liver.

[294] Colombo C., Battezzati P.M., Crosignani A., Morabito A., Costantini D., Padoan R., Giunta A. (2002). Liver disease in cystic fibrosis: A prospective study on incidence, risk factors, and outcome. Hepatology.

[295] Strandvik B. (2006). Care of patients with cystic fibrosis. Treatment, screening and clinical outcome. Ann. Nestlé.

[296] Colombo C., Alicandro G., Oliver M., Lewindon P.J., Ramm G.A., Ooi C.Y., Alghisi F., Kashirskaya N., Kondratyeva E., Corti F. (2022). Ursodeoxycholic acid and liver disease associated with cystic fibrosis: A multicenter cohort study. J. Cyst. Fibros..

[297] Witters P., Libbrecht L., Roskams T., Boeck K.D., Dupont L., Proesmans M., Vermeulen F., Strandvik B., Lindblad A., Stéphenne X. (2011). Noncirrhotic presinusoidal portal hypertension is common in cystic fibrosis-associated liver disease. Hepatology.

[298] Witters P., Libbrecht L., Roskams T., De Boeck K., Dupont L., Proesmans M., Vermeulen F., Maleux G., Monbaliu D., Pirenne J., Cassiman D. (2017). Liver disease in cystic fibrosis presents as non-cirrhotic portal hypertension. J. Cyst. Fibros..

[299] Bryan R.M., You J., Golding E.M., Marrelli S.P. (2005). Endothelium-derived hyperpolarizing factor: a cousin to nitric oxide and prostacyclin. Anesthesiology.

[300] Mallick R., Duttaroy A.K. (2022). Modulation of endothelium function by fatty acids. Mol. Cell. Biochem..

[301] Nickl R., Hauser S., Pietzsch J., Richter T. (2023). Significance of Pulmonary Endothelial Injury and the Role of Cyclooxygenase-2 and Prostanoid Signaling. Bioengineering (Basel).

[302] Strandvik B., Hjelte L., Gabrielsson N., Glaumann H. (1988). Sclerosing cholangitis in cystic fibrosis. Scand. J. Gastroenterol. Suppl..

[303] Durieu I., Pellet O., Simonot L., Durupt S., Bellon G., Durand D.V., Minh V.A. (1999). Sclerosing cholangitis in adults with cystic fibrosis: a magnetic resonance cholangiographic prospective study. J. Hepatol..

[304] Lindblad A., Hultcrantz R., Strandvik B. (1992). Bile-duct destruction and collagen deposition: a prominent ultrastructural feature of the liver in cystic fibrosis. Hepatology.

[305] Hultcrantz R., Mengarelli S., Strandvik B. (1986). Morphological findings in the liver of children with cystic fibrosis: a light and electron microscopical study. Hepatology.

[306] Sheth S., Shea J.C., Bishop M.D., Chopra S., Regan M.M., Malmberg E., Walker C., Ricci R., Tsui L.C., Durie P.R. (2003). Increased prevalence of CFTR mutations and variants and decreased chloride secretion in primary sclerosing cholangitis. Hum. Genet..

[307] Hough N.E., Chapman S.J., Flight W.G. (2020). Gastrointestinal malignancy in cystic fibrosis. Paediatr. Respir. Rev..

[308] Neglia J.P., FitzSimmons S.C., Maisonneuve P., Schöni M.H., Schöni-Affolter F., Corey M., Lowenfels A.B. (1995). The risk of cancer among patients with cystic fibrosis. Cystic Fibrosis and Cancer Study Group. N. Engl. J. Med..

[309] Eklund A., Norman A., Strandvik B. (1980). Excretion of bile acids in healthy children and children with cystic fibrosis. Scand. J. Clin. Lab. Invest..

[310] van de Peppel I.P., Bodewes F., Verkade H.J., Jonker J.W. (2019). Bile acid homeostasis in gastrointestinal and metabolic complications of cystic fibrosis. J. Cyst. Fibros..

[311] Strandvik B., Einarsson K., Lindblad A., Angelin B. (1996). Bile acid kinetics and biliary lipid composition in cystic fibrosis. J. Hepatol..

[312] Arborgh B., Eklund A., Norman A., Strandvik B. (1980). Urinary bile acid excretion in correlation to liver histopathology in cystic fibrosis. Scand. J. Gastroenterol..

[313] Strandvik B., Samuelson K. (1985). Fasting serum bile acid levels in relation to liver histopathology in cystic fibrosis. Scand. J. Gastroenterol..

[314] Drzymała-Czyż S., Dziedzic K., Szwengiel A., Krzyżanowska-Jankowska P., Nowak J.K., Nowicka A., Aringazina R., Drzymała S., Kashirskaya N., Walkowiak J. (2022). Serum bile acids in cystic fibrosis patients - glycodeoxycholic acid as a potential marker of liver disease. Dig. Liver Dis..

[315] Lai S., Mazzaferro S., Mitterhofer A.P., Bonci E., Marotta P.G., Pelligra F., Murciano M., Celani C., Troiani P., Cimino G., Palange P. (2019). Renal involvement and metabolic alterations in adults patients affected by cystic fibrosis. J. Transl. Med..

[316] Strandvik B. (1982). Renal disturbance in cystic fibrosis. Lancet.

[317] Berg U., Kusoffsky E., Strandvik B. (1982). Renal function in cystic fibrosis with special reference to the renal sodium handling. Acta Paediatr. Scand..

[318] Strandvik B., Berg U., Kallner A., Kusoffsky E. (1989). Effect on renal function of essential fatty acid supplementation in cystic fibrosis. J. Pediatr..

[319] Declercq D., Van Braeckel E., Marchand S., Van Daele S., Van Biervliet S. (2020). Sodium Status and Replacement in Children and Adults Living with Cystic Fibrosis: A Narrative Review. J. Acad. Nutr. Diet..

[320] Strandvik B., Bergström K., Broberger O., Freyschuss U., Plantin L.O. (1981). Total exchangable sodium in cystic fibrosis. Monogr. Paediatr..

[321] Knepper C., Ellemunter H., Eder J., Niedermayr K., Haerter B., Hofer P., Scholl-Bürgi S., Müller T., Heinz-Erian P. (2016). Low sodium status in cystic fibrosis-as assessed by calculating fractional Na(+) excretion-is associated with decreased growth parameters. J. Cyst. Fibros..

[322] Rossier B.C. (2014). Epithelial sodium channel (ENaC) and the control of blood pressure. Curr. Opin. Pharmacol..

[323] Hill W.G., Butterworth M.B., Wang H., Edinger R.S., Lebowitz J., Peters K.W., Frizzell R.A., Johnson J.P. (2007). The epithelial sodium channel (ENaC) traffics to apical membrane in lipid rafts in mouse cortical collecting duct cells. J. Biol. Chem..

[324] Strandvik B. (2021). Is the ENaC Dysregulation in CF an Effect of Protein-Lipid Interaction in the Membranes?. Int. J. Mol. Sci..

[325] Bhalla V., Hallows K.R. (2008). Mechanisms of ENaC regulation and clinical implications. J. Am. Soc. Nephrol..

[326] Soodvilai S., Jia Z., Fongsupa S., Chatsudthipong V., Yang T. (2012). Liver X receptor agonists decrease ENaC-mediated sodium transport in collecting duct cells. Am. J. Physiol. Ren. Physiol..

[327] De Sutter P.J., Van Haeverbeke M., Van Braeckel E., Van Biervliet S., Van Bocxlaer J., Vermeulen A., Gasthuys E. (2022). Altered intravenous drug disposition in people living with cystic fibrosis: A meta-analysis integrating top-down and bottom-up data. CPT Pharmacometrics Syst. Pharmacol..

[328] Stenvinkel P., Hjelte L., Alván G., Hedman A., Hultman E., Strandvik B. (1991). Decreased renal clearance of sodium in cystic fibrosis. Acta Paediatr. Scand..

[329] Akkerman-Nijland A.M., Akkerman O.W., Grasmeijer F., Hagedoorn P., Frijlink H.W., Rottier B.L., Koppelman G.H., Touw D.J. (2021). The pharmacokinetics of antibiotics in cystic fibrosis. Expet Opin. Drug Metabol. Toxicol..

[330] Alván G., Beermann B., Hjelte L., Lind M., Lindholm A., Strandvik B. (1988). Increased nonrenal clearance and increased diuretic efficiency of furosemide in cystic fibrosis. Clin. Pharmacol. Ther..

[331] Hedman A., Alván G., Strandvik B., Arvidsson A. (1990). Increased renal clearance of cefsulodin due to higher glomerular filtration rate in cystic fibrosis. Clin. Pharmacokinet..

[332] Hjelte L., Larsson M., Alvestrand A., Malmborg A.S., Strandvik B. (1990). Renal function in rats with essential fatty acid deficiency. Clin. Sci. (Lond.).

[333] Caligiuri S.P., Love K., Winter T., Gauthier J., Taylor C.G., Blydt-Hansen T., Zahradka P., Aukema H.M. (2013). Dietary linoleic acid and α-linolenic acid differentially affect renal oxylipins and phospholipid fatty acids in diet-induced obese rats. J. Nutr..

[334] Leng S., Winter T., Aukema H.M. (2017). Dietary LA and sex effects on oxylipin profiles in rat kidney, liver, and serum differ from their effects on PUFAs. J. Lipid Res..

[335] Hjelte L., Melin T., Nilsson A., Strandvik B. (1990). Absorption and metabolism of [3H]arachidonic and [14C]linoleic acid in essential fatty acid-deficient rats. Am. J. Physiol..

[336] Lefkowith J.B., Flippo V., Sprecher H., Needleman P. (1985). Paradoxical conservation of cardiac and renal arachidonate content in essential fatty acid deficiency. J. Biol. Chem..

[337] Wang S., Meng F., Xu J., Gu Y. (2009). Effects of lipids on ENaC activity in cultured mouse cortical collecting duct cells. J. Membr. Biol..

[338] Tirouvanziam R., Khazaal I., Péault B. (2002). Primary inflammation in human cystic fibrosis small airways. Am. J. Physiol. Lung Cell Mol. Physiol..

[339] Balough K., McCubbin M., Weinberger M., Smits W., Ahrens R., Fick R. (1995). The relationship between infection and inflammation in the early stages of lung disease from cystic fibrosis. Pediatr. Pulmonol..

[340] Keiser N.W., Birket S.E., Evans I.A., Tyler S.R., Crooke A.K., Sun X., Zhou W., Nellis J.R., Stroebele E.K., Chu K.K. (2015). Defective innate immunity and hyperinflammation in newborn cystic fibrosis transmembrane conductance regulator-knockout ferret lungs. Am. J. Respir. Cell Mol. Biol..

[341] Gilljam H., Strandvik B., Ellin A., Wiman L.G. (1986). Increased mole fraction of arachidonic acid in bronchial phospholipids in patients with cystic fibrosis. Scand. J. Clin. Lab. Invest..

[342] Carlstedt-Duke J., Brönnegård M., Strandvik B. (1986). Pathological regulation of arachidonic acid release in cystic fibrosis: the putative basic defect. Proc. Natl. Acad. Sci. USA.

[343] Keen C., Olin A.C., Edentoft A., Gronowitz E., Strandvik B. (2007). Airway nitric oxide in patients with cystic fibrosis is associated with pancreatic function, Pseudomonas infection, and polyunsaturated fatty acids. Chest.

[344] Keen C., Olin A.C., Eriksson S., Ekman A., Lindblad A., Basu S., Beermann C., Strandvik B. (2010). Supplementation with fatty acids influences the airway nitric oxide and inflammatory markers in patients with cystic fibrosis. J. Pediatr. Gastroenterol. Nutr..

[345] Witters P., Dupont L., Vermeulen F., Proesmans M., Cassiman D., Wallemacq P., De Boeck K. (2013). Lung transplantation in cystic fibrosis normalizes essential fatty acid profiles. J. Cyst. Fibros..

[346] Chase H.P., Cotton E.K., Elliott R.B. (1979). Intravenous linoleic acid supplementation in children with cystic fibrosis. Pediatrics.

[347] Craig-Schmidt M.C., Faircloth S.A., Teer P.A., Weete J.D., Wu C.Y. (1986). The essential fatty acid deficient chicken as a model for cystic fibrosis. Am. J. Clin. Nutr..

[348] Knudsen P.K., Olesen H.V., Høiby N., Johannesson M., Karpati F., Laerum B.N., Meyer P., Pressler T., Lindblad A. (2009). Differences in prevalence and treatment of Pseudomonas aeruginosa in cystic fibrosis centres in Denmark, Norway and Sweden. J. Cyst. Fibros..

[349] Hansen C.R., Gilljam M., Olesen H.V., Høiby N., Karpati F., Johansson E., Krantz C., Skov M., Pressler T., Lindblad A. (2021). Maintaining normal lung function in children with cystic fibrosis is possible with aggressive treatment regardless of Pseudomonas aeruginosa infections. Acta Paediatr..

[350] Jaganathan D., Bruscia E.M., Kopp B.T. (2022). Emerging Concepts in Defective Macrophage Phagocytosis in Cystic Fibrosis. Int. J. Mol. Sci..

[351] Liu N.M., Miyashita L., Sanak M., Barratt B., Grigg J. (2021). Prostaglandin E(2) and phagocytosis of inhaled particulate matter by airway macrophages in cystic fibrosis. J. Cyst. Fibros..

[352] Zhang S., Shrestha C.L., Robledo-Avila F., Jaganathan D., Wisniewski B.L., Brown N., Pham H., Carey K., Amer A.O., Hall-Stoodley L. (2023). Cystic fibrosis macrophage function and clinical outcomes after elexacaftor/tezacaftor/ivacaftor. Eur. Respir. J..

[353] Paccou J., Zeboulon N., Combescure C., Gossec L., Cortet B. (2010). The prevalence of osteoporosis, osteopenia, and fractures among adults with cystic fibrosis: a systematic literature review with meta-analysis. Calcif. Tissue Int..

[354] Gronowitz E., Garemo M., Lindblad A., Mellström D., Strandvik B. (2003). Decreased bone mineral density in normal-growing patients with cystic fibrosis. Acta Paediatr..

[355] Garemo M., Strandvik B. (1999). Nutrition in Cystic Fibrosis. Scand. J. Nutr..

[356] Gronowitz E., Mellström D., Strandvik B. (2004). Normal annual increase of bone mineral density during two years in patients with cystic fibrosis. Pediatrics.

[357] Gronowitz E., Mellström D., Strandvik B. (2006). Serum phospholipid fatty acid pattern is associated with bone mineral density in children, but not adults, with cystic fibrosis. Br. J. Nutr..

[358] Gronowitz E., Lorentzon M., Ohlsson C., Mellström D., Strandvik B. (2008). Docosahexaenoic acid is associated with endosteal circumference in long bones in young males with cystic fibrosis. Br. J. Nutr..

[359] Stagi S., Cavalli L., Iurato C., Seminara S., Brandi M.L., de Martino M. (2013). Bone metabolism in children and adolescents: main characteristics of the determinants of peak bone mass. Clin. Cases Miner. Bone Metab..

[360] Lau B.Y., Cohen D.J., Ward W.E., Ma D.W. (2013). Investigating the role of polyunsaturated fatty acids in bone development using animal models. Molecules.

[361] Watkins B.A., Li Y., Lippman H.E., Feng S. (2003). Modulatory effect of omega-3 polyunsaturated fatty acids on osteoblast function and bone metabolism. Prostaglandins Leukot. Essent. Fatty Acids.

[362] Wauquier F., Léotoing L., Philippe C., Spilmont M., Coxam V., Wittrant Y. (2015). Pros and cons of fatty acids in bone biology. Prog. Lipid Res..

[363] Watkins B.A., Lippman H.E., Le Bouteiller L., Li Y., Seifert M.F. (2001). Bioactive fatty acids: role in bone biology and bone cell function. Prog. Lipid Res..

[364] Harada S.I., Balena R., Rodan G.A., Rodan S.B. (1995). The role of prostaglandins in bone formation. Connect. Tissue Res..

[365] Garemo M., Sundh V., Mellström D., Strandvik B. (2024). Serum phospholipid fatty acids are associated with bone mass in healthy 4-years-old children. Prostaglandins Leukot. Essent. Fatty Acids.

[366] Eriksson S., Mellström D., Strandvik B. (2009). Fatty acid pattern in serum is associated with bone mineralisation in healthy 8-year-old children. Br. J. Nutr..

[367] Smyth R.L., Rayner O. (2014). Oral calorie supplements for cystic fibrosis. Cochrane Database Syst. Rev..

[368] Aldámiz-Echevarría L., Prieto J.A., Andrade F., Elorz J., Sojo A., Lage S., Sanjurjo P., Vázquez C., Rodríguez-Soriano J. (2009). Persistence of essential fatty acid deficiency in cystic fibrosis despite nutritional therapy. Pediatr. Res..

[369] Lloyd-Still J.D., Bibus D.M., Powers C.A., Johnson S.B., Holman R.T. (1996). Essential fatty acid deficiency and predisposition to lung disease in cystic fibrosis. Acta Paediatr..

[370] Walkowiak J., Przyslawski J. (2003). Five-year prospective analysis of dietary intake and clinical status in malnourished cystic fibrosis patients. J. Hum. Nutr. Diet..

[371] Simon M., Dalle Molle R., Silva F.M., Rodrigues T.W., Feldmann M., Forte G.C., Marostica P.J.C. (2020). Antioxidant Micronutrients and Essential Fatty Acids Supplementation on Cystic Fibrosis Outcomes: A Systematic Review. J. Acad. Nutr. Diet..

[372] Wilschanski M., Munck A., Carrion E., Cipolli M., Collins S., Colombo C., Declercq D., Hatziagorou E., Hulst J., Kalnins D. (2024). ESPEN-ESPGHAN-ECFS guideline on nutrition care for cystic fibrosis. Clin. Nutr..

[373] Friesen R.W., Innis S.M. (2010). Linoleic acid is associated with lower long-chain n-6 and n-3 fatty acids in red blood cell lipids of Canadian pregnant women. Am. J. Clin. Nutr..

[374] Zaman M.M., Martin C.R., Andersson C., Bhutta A.Q., Cluette-Brown J.E., Laposata M., Freedman S.D. (2010). Linoleic acid supplementation results in increased arachidonic acid and eicosanoid production in CF airway cells and in cftr-/- transgenic mice. Am. J. Physiol. Lung Cell Mol. Physiol..

[375] Vij N. (2010). Linoleic acid supplement in cystic fibrosis: friend or foe?. Am. J. Physiol. Lung Cell Mol. Physiol..

[376] Blomquist M., Freyschuss U., Wiman L.G., Strandvik B. (1986). Physical activity and self treatment in cystic fibrosis. Arch. Dis. Child..

[377] Strandvik B., Hjelte L., Malmborg A.S., Widén B. (1992). Home intravenous antibiotic treatment of patients with cystic fibrosis. Acta Paediatr..

[378] Strandvik B. (1988). Antibiotic therapy of pulmonary infections in cystic fibrosis. Dosage schedules and duration of treatment. Chest.

[379] Oliver C., Jahnke N. (2011). Omega-3 fatty acids for cystic fibrosis. Cochrane Database Syst. Rev..

[380] Van Biervliet S., Devos M., Delhaye T., Van Biervliet J.P., Robberecht E., Christophe A. (2008). Oral DHA supplementation in DeltaF508 homozygous cystic fibrosis patients. Prostaglandins Leukot. Essent. Fatty Acids.

[381] Alicandro G., Faelli N., Gagliardini R., Santini B., Magazzù G., Biffi A., Risé P., Galli C., Tirelli A.S., Loi S. (2013). A randomized placebo-controlled study on high-dose oral algal docosahexaenoic acid supplementation in children with cystic fibrosis. Prostaglandins Leukot. Essent. Fatty Acids.

[382] López-Neyra A., Suárez L., Muñoz M., de Blas A., Ruiz de Valbuena M., Garriga M., Calvo J., Ribes C., Girón Moreno R., Máiz L. (2020). Long-term docosahexaenoic acid (DHA) supplementation in cystic fibrosis patients: a randomized, multi-center, double-blind, placebo-controlled trial. Prostaglandins Leukot. Essent. Fatty Acids.

[383] Ollero M., Astarita G., Guerrera I.C., Sermet-Gaudelus I., Trudel S., Piomelli D., Edelman A. (2011). Plasma lipidomics reveals potential prognostic signatures within a cohort of cystic fibrosis patients. J. Lipid Res..

[384] Eickmeier O., Fussbroich D., Mueller K., Serve F., Smaczny C., Zielen S., Schubert R. (2017). Pro-resolving lipid mediator Resolvin D1 serves as a marker of lung disease in cystic fibrosis. PLoS One.

[385] Durieu I., Vericel E., Guichardant D., Roth H., Steghens J.P., Drai J., Josserand R.N., Fontaine E., Lagarde M., Bellon G. (2007). Fatty acids platelets and oxidative markers following intravenous n-3 fatty acids administration in cystic fibrosis: An open pilot observational study. J. Cyst. Fibros..

[386] Olveira G., Olveira C., Acosta E., Espíldora F., Garrido-Sánchez L., García-Escobar E., Rojo-Martínez G., Gonzalo M., Soriguer F. (2010). Fatty acid supplements improve respiratory, inflammatory and nutritional parameters in adults with cystic fibrosis. Arch. Bronconeumol..

[387] Dyall S.C., Balas L., Bazan N.G., Brenna J.T., Chiang N., da Costa Souza F., Dalli J., Durand T., Galano J.M., Lein P.J. (2022). Polyunsaturated fatty acids and fatty acid-derived lipid mediators: Recent advances in the understanding of their biosynthesis, structures, and functions. Prog. Lipid Res..

[388] Morin C., Cantin A.M., Rousseau É., Sirois M., Sirois C., Rizcallah E., Fortin S. (2015). Proresolving Action of Docosahexaenoic Acid Monoglyceride in Lung Inflammatory Models Related to Cystic Fibrosis. Am. J. Respir. Cell Mol. Biol..

[389] Ferrucci L., Cherubini A., Bandinelli S., Bartali B., Corsi A., Lauretani F., Martin A., Andres-Lacueva C., Senin U., Guralnik J.M. (2006). Relationship of plasma polyunsaturated fatty acids to circulating inflammatory markers. J. Clin. Endocrinol. Metab..

[390] Kalish B.T., Le H.D., Fitzgerald J.M., Wang S., Seamon K., Gura K.M., Gronert K., Puder M. (2013). Intravenous fish oil lipid emulsion promotes a shift toward anti-inflammatory proresolving lipid mediators. Am. J. Physiol. Gastrointest. Liver Physiol..

[391] Al-Turkmani M.R., Andersson C., Alturkmani R., Katrangi W., Cluette-Brown J.E., Freedman S.D., Laposata M. (2008). A mechanism accounting for the low cellular level of linoleic acid in cystic fibrosis and its reversal by DHA. J. Lipid Res..

[392] O'Connor M.G., Thomsen K., Brown R.F., Laposata M., Seegmiller A. (2016). Elevated prostaglandin E metabolites and abnormal plasma fatty acids at baseline in pediatric cystic fibrosis patients: a pilot study. Prostaglandins Leukot. Essent. Fatty Acids.

[393] Cottrill K.A., Farinha C.M., McCarty N.A. (2020). The bidirectional relationship between CFTR and lipids. Commun. Biol..

[394] Anderson M.P., Welsh M.J. (1990). Fatty acids inhibit apical membrane chloride channels in airway epithelia. Proc. Natl. Acad. Sci. USA.

[395] Linsdell P. (2000). Inhibition of cystic fibrosis transmembrane conductance regulator chloride channel currents by arachidonic acid. Can. J. Physiol. Pharmacol..

[396] Zhou J.J., Linsdell P. (2007). Molecular mechanism of arachidonic acid inhibition of the CFTR chloride channel. Eur. J. Pharmacol..

[397] Ciardo M.G., Ferrer-Montiel A. (2017). Lipids as central modulators of sensory TRP channels. Biochim. Biophys. Acta Biomembr..

[398] Seo J., Barhoumi R., Johnson A.E., Lupton J.R., Chapkin R.S. (2006). Docosahexaenoic acid selectively inhibits plasma membrane targeting of lipidated proteins. Faseb. J..

[399] Laganowsky A., Reading E., Allison T.M., Ulmschneider M.B., Degiacomi M.T., Baldwin A.J., Robinson C.V. (2014). Membrane proteins bind lipids selectively to modulate their structure and function. Nature.

[400] Koshy C., Ziegler C. (2015). Structural insights into functional lipid-protein interactions in secondary transporters. Biochim. Biophys. Acta.

[401] Weingarth M., Prokofyev A., van der Cruijsen E.A., Nand D., Bonvin A.M., Pongs O., Baldus M. (2013). Structural determinants of specific lipid binding to potassium channels. J. Am. Chem. Soc..

[402] Lee A.G. (2003). Lipid-protein interactions in biological membranes: a structural perspective. Biochim. Biophys. Acta.

[403] Keenan T.W., Morré D.J. (1970). Phospholipid class and fatty acid composition of golgi apparatus isolated from rat liver and comparison with other cell fractions. Biochemistry.

[404] Langelier B., Linard A., Bordat C., Lavialle M., Heberden C. (2010). Long chain-polyunsaturated fatty acids modulate membrane phospholipid composition and protein localization in lipid rafts of neural stem cell cultures. J. Cell. Biochem..

[405] Eidelman O., BarNoy S., Razin M., Zhang J., McPhie P., Lee G., Huang Z., Sorscher E.J., Pollard H.B. (2002). Role for phospholipid interactions in the trafficking defect of Delta F508-CFTR. Biochemistry.

[406] Hildebrandt E., Khazanov N., Kappes J.C., Dai Q., Senderowitz H., Urbatsch I.L. (2017). Specific stabilization of CFTR by phosphatidylserine. Biochim. Biophys. Acta Biomembr..

[407] Strandvik B., Brönnegård M., Gilljam H., Carlstedt-Duke J. (1988). Relation between defective regulation of arachidonic acid release and symptoms in cystic fibrosis. Scand. J. Gastroenterol. Suppl..

[408] Miele L., Cordella-Miele E., Xing M., Frizzell R., Mukherjee A.B. (1997). Cystic fibrosis gene mutation (deltaF508) is associated with an intrinsic abnormality in Ca2+-induced arachidonic acid release by epithelial cells. DNA Cell Biol..

[409] Levistre R., Lemnaouar M., Rybkine T., Béréziat G., Masliah J. (1993). Increase of bradykinin-stimulated arachidonic acid release in a delta F508 cystic fibrosis epithelial cell line. Biochim. Biophys. Acta.

[410] Berguerand M., Klapisz E., Thomas G., Humbert L., Jouniaux A.M., Olivier J.L., Béréziat G., Masliah J. (1997). Differential stimulation of cytosolic phospholipase A2 by bradykinin in human cystic fibrosis cell lines. Am. J. Respir. Cell Mol. Biol..

[411] Seegmiller A.C. (2014). Abnormal unsaturated fatty acid metabolism in cystic fibrosis: biochemical mechanisms and clinical implications. Int. J. Mol. Sci..

[412] Umunakwe O.C., Seegmiller A.C. (2014). Abnormal n-6 fatty acid metabolism in cystic fibrosis is caused by activation of AMP-activated protein kinase. J. Lipid Res..

[413] Silwal P., Kim J.K., Yuk J.M., Jo E.K. (2018). AMP-Activated Protein Kinase and Host Defense against Infection. Int. J. Mol. Sci..

[414] Bensalem N., Ventura A.P., Vallée B., Lipecka J., Tondelier D., Davezac N., Dos Santos A., Perretti M., Fajac A., Sermet-Gaudelus I. (2005). Down-regulation of the anti-inflammatory protein annexin A1 in cystic fibrosis knock-out mice and patients. Mol. Cell. Proteomics.

[415] Croxtall J.D., Gilroy D.W., Solito E., Choudhury Q., Ward B.J., Buckingham J.C., Flower R.J. (2003). Attenuation of glucocorticoid functions in an Anx-A1-/- cell line. Biochem. J..

[416] Roviezzo F., Getting S.J., Paul-Clark M.J., Yona S., Gavins F.N., Perretti M., Hannon R., Croxtall J.D., Buckingham J.C., Flower R.J. (2002). The annexin-1 knockout mouse: what it tells us about the inflammatory response. J. Physiol. Pharmacol..

[417] Yona S., Buckingham J.C., Perretti M., Flower R.J. (2004). Stimulus-specific defect in the phagocytic pathways of annexin 1 null macrophages. Br. J. Pharmacol..

[418] Yona S., Ward B., Buckingham J.C., Perretti M., Flower R.J. (2005). Macrophage biology in the Anx-A1-/- mouse. Prostaglandins Leukot. Essent. Fatty Acids.

[419] Arur S., Uche U.E., Rezaul K., Fong M., Scranton V., Cowan A.E., Mohler W., Han D.K. (2003). Annexin I is an endogenous ligand that mediates apoptotic cell engulfment. Dev. Cell.

[420] Yu C., Zhang L. (2022). Methylprednisolone up-regulates annexin A1 (ANXA1) to inhibit the inflammation, apoptosis and oxidative stress of cigarette smoke extract (CSE)-induced bronchial epithelial cells, a chronic obstructive pulmonary disease *in vitro* model, through the formyl peptide receptor 2 (FPR2) receptors and the adenosine 5'-monophosphate (AMP)-activated protein kinase (AMPK) pathway. Bioengineered.

[421] Borthwick L.A., Riemen C., Goddard C., Colledge W.H., Mehta A., Gerke V., Muimo R. (2008). Defective formation of PKA/CnA-dependent annexin 2-S100A10/CFTR complex in DeltaF508 cystic fibrosis cells. Cell. Signal..

[422] Roulin K., Hagens G., Hotz R., Saurat J.H., Veerkamp J.H., Siegenthaler G. (1999). The fatty acid-binding heterocomplex FA-p34 formed by S100A8 and S100A9 is the major fatty acid carrier in neutrophils and translocates from the cytosol to the membrane upon stimulation. Exp. Cell Res..

[423] Tirkos S., Newbigging S., Nguyen V., Keet M., Ackerley C., Kent G., Rozmahel R.F. (2006). Expression of S100A8 correlates with inflammatory lung disease in congenic mice deficient of the cystic fibrosis transmembrane conductance regulator. Respir. Res..

[424] Chatterjee B.E., Yona S., Rosignoli G., Young R.E., Nourshargh S., Flower R.J., Perretti M. (2005). Annexin 1-deficient neutrophils exhibit enhanced transmigration *in vivo* and increased responsiveness *in vitro*. J. Leukoc. Biol..

[425] Chap H., Fauvel J., Gassama-Diagne A., Ragab-Thomas J., Simon M.-F. (1991). Une homologie frappante entre le CFTR et les annexines. Medicine/Science.

[426] D'Orazio S., Mattoscio D. (2024). Dysregulation of the Arachidonic Acid Pathway in Cystic Fibrosis: Implications for Chronic Inflammation and Disease Progression. Pharmaceuticals.

[427] Myerburg M.M., King J.D., Oyster N.M., Fitch A.C., Magill A., Baty C.J., Watkins S.C., Kolls J.K., Pilewski J.M., Hallows K.R. (2010). AMPK agonists ameliorate sodium and fluid transport and inflammation in cystic fibrosis airway epithelial cells. Am. J. Respir. Cell Mol. Biol..

[428] Hallows K.R., Fitch A.C., Richardson C.A., Reynolds P.R., Clancy J.P., Dagher P.C., Witters L.A., Kolls J.K., Pilewski J.M. (2006). Up-regulation of AMP-activated kinase by dysfunctional cystic fibrosis transmembrane conductance regulator in cystic fibrosis airway epithelial cells mitigates excessive inflammation. J. Biol. Chem..

[429] Kunzelmann K., Schreiber R., Nitschke R., Mall M. (2000). Control of epithelial Na+ conductance by the cystic fibrosis transmembrane conductance regulator. Pflügers Archiv.

[430] Nagel G., Barbry P., Chabot H., Brochiero E., Hartung K., Grygorczyk R. (2005). CFTR fails to inhibit the epithelial sodium channel ENaC expressed in Xenopus laevis oocytes. J. Physiol..

[431] Berdiev B.K., Qadri Y.J., Benos D.J. (2009). Assessment of the CFTR and ENaC association. Mol. Biosyst..

[432] Lazrak A., Jurkuvenaite A., Chen L., Keeling K.M., Collawn J.F., Bedwell D.M., Matalon S. (2011). Enhancement of alveolar epithelial sodium channel activity with decreased cystic fibrosis transmembrane conductance regulator expression in mouse lung. Am. J. Physiol. Lung Cell Mol. Physiol..

[433] Enuka Y., Hanukoglu I., Edelheit O., Vaknine H., Hanukoglu A. (2012). Epithelial sodium channels (ENaC) are uniformly distributed on motile cilia in the oviduct and the respiratory airways. Histochem. Cell Biol..

[434] Feraille E., Dizin E. (2016). Coordinated Control of ENaC and Na+,K+-ATPase in Renal Collecting Duct. J. Am. Soc. Nephrol..

[435] Sigström L., Strandvik B. (1992). Erythrocyte sodium-potassium transport in cystic fibrosis. Pediatr. Res..

[436] Mall M.A. (2020). ENaC inhibition in cystic fibrosis: potential role in the new era of CFTR modulator therapies. Eur. Respir. J..

[437] Mailhot G., Rabasa-Lhoret R., Moreau A., Berthiaume Y., Levy E. (2010). CFTR depletion results in changes in fatty acid composition and promotes lipogenesis in intestinal Caco 2/15 cells. PLoS One.

[438] Raksaseri P., Chatsudthipong V., Muanprasat C., Soodvilai S. (2013). Activation of liver X receptors reduces CFTR-mediated Cl(-) transport in kidney collecting duct cells. Am. J. Physiol. Ren. Physiol..

[439] Joseph S.B., Castrillo A., Laffitte B.A., Mangelsdorf D.J., Tontonoz P. (2003). Reciprocal regulation of inflammation and lipid metabolism by liver X receptors. Nat Med.

[440] Bensinger S.J., Bradley M.N., Joseph S.B., Zelcer N., Janssen E.M., Hausner M.A., Shih R., Parks J.S., Edwards P.A., Jamieson B.D., Tontonoz P. (2008). LXR signaling couples sterol metabolism to proliferation in the acquired immune response. Cell.

[441] Hong C., Tontonoz P. (2008). Coordination of inflammation and metabolism by PPAR and LXR nuclear receptors. Curr. Opin. Genet. Dev..

[442] Castrillo A., Joseph S.B., Vaidya S.A., Haberland M., Fogelman A.M., Cheng G., Tontonoz P. (2003). Crosstalk between LXR and toll-like receptor signaling mediates bacterial and viral antagonism of cholesterol metabolism. Mol. Cell.

[443] Ishibashi M., Varin A., Filomenko R., Lopez T., Athias A., Gambert P., Blache D., Thomas C., Gautier T., Lagrost L., Masson D. (2013). Liver x receptor regulates arachidonic acid distribution and eicosanoid release in human macrophages: a key role for lysophosphatidylcholine acyltransferase 3. Arterioscler. Thromb. Vasc. Biol..

[444] Sampath H., Ntambi J.M. (2005). Polyunsaturated fatty acid regulation of genes of lipid metabolism. Annu. Rev. Nutr..

[445] Veit G., Avramescu R.G., Chiang A.N., Houck S.A., Cai Z., Peters K.W., Hong J.S., Pollard H.B., Guggino W.B., Balch W.E. (2016). From CFTR biology toward combinatorial pharmacotherapy: expanded classification of cystic fibrosis mutations. Mol. Biol. Cell.

[446] Raraigh K.S., Paul K.C., Goralski J.L., Worthington E.N., Faino A.V., Sciortino S., Wang Y., Aksit M.A., Ling H., Osorio D.L. (2022). CFTR bearing variant p.Phe312del exhibits function inconsistent with phenotype and negligible response to ivacaftor. JCI Insight.

[447] Lee A.G. (2011). Biological membranes: the importance of molecular detail. Trends Biochem. Sci..

[448] Escribá P.V. (2017). Membrane-lipid therapy: A historical perspective of membrane-targeted therapies - From lipid bilayer structure to the pathophysiological regulation of cells. Biochim. Biophys. Acta Biomembr..

